# Circulating isoflavone and lignan concentrations and prostate cancer risk: a meta‐analysis of individual participant data from seven prospective studies including 2,828 cases and 5,593 controls

**DOI:** 10.1002/ijc.31640

**Published:** 2018-09-29

**Authors:** Aurora Perez‐Cornago, Paul N. Appleby, Heiner Boeing, Leire Gil, Cecilie Kyrø, Fulvio Ricceri, Neil Murphy, Antonia Trichopoulou, Konstantinos K. Tsilidis, Kay‐Tee Khaw, Robert N. Luben, Randi E Gislefoss, Hilde Langseth, Isabel Drake, Emily Sonestedt, Peter Wallström, Pär Stattin, Anders Johansson, Rikard Landberg, Lena Maria Nilsson, Kotaro Ozasa, Akiko Tamakoshi, Kazuya Mikami, Tatsuhiko Kubo, Norie Sawada, Shoichiro Tsugane, Timothy J. Key, Naomi E. Allen, Ruth C. Travis

**Affiliations:** ^1^ Cancer Epidemiology Unit, Nuffield Department of Population Health University of Oxford Oxford United Kingdom; ^2^ Department of Epidemiology German Institute of Human Nutrition Potsdam‐Rehbrücke Nuthetal Germany; ^3^ Public Health Division of Gipuzkoa‐BIODONOSTIA Basque Regional Health Department San Sebastian Spain; ^4^ CIBER of Epidemiology and Public Health Madrid Spain; ^5^ Danish Cancer Society Research Center, Strandboulevarden 49 Copenhagen Denmark; ^6^ Department of Clinical and Biological Sciences University of Turin Turin Italy; ^7^ Unit of Epidemiology Regional Health Service ASL TO3 Grugliasco Italy; ^8^ Section of Nutrition and Metabolism International Agency for Research on Cancer Lyon France; ^9^ Hellenic Health Foundation Athens Greece; ^10^ Department of Epidemiology and Biostatistics School of Public Health, Imperial College London London United Kingdom; ^11^ Department of Hygiene and Epidemiology University of Ioannina School of Medicine Ioannina Greece; ^12^ Department of Public Health and Primary Care University of Cambridge Cambridge United Kingdom; ^13^ Department of Research Cancer Registry of Norway Oslo Norway; ^14^ Department of Clinical Sciences in Malmö Lund University Malmö Sweden; ^15^ Clinical Research Centre Skåne University Hospital Malmö Sweden; ^16^ Department of Surgical Sciences Uppsala University Uppsala Sweden; ^17^ Nutritional Research and Molecular Periodontology Umeå University Umeö Sweden; ^18^ Department of Biology and Biological Engineering Food and Nutrition Science, Chalmers University of Technology Gothenburg Sweden; ^19^ Department of Public Health and Clinical Medicine Nutritional Research, Umeå University Umeå Sweden; ^20^ Arctic Research Centre, Umeå University Umeå Sweden; ^21^ Department of Epidemiology Radiation Effects Research Foundation Minami‐ku Hiroshima Japan; ^22^ Department of Public Health Hokkaido University Graduate School of Medicine Kita‐ku Sapporo Japan; ^23^ Department of Urology Kyoto Prefectural University of Medicine Graduate School of Medical Science Kamikgyo‐ku Kyoto Japan; ^24^ Department of Preventive Medicine and Community Health University of Occupational and Environmental Health Yahatanishi‐ku Kitakyushu Japan; ^25^ Epidemiology and Prevention Group Center for Public Health Sciences, National Cancer Center Tokyo Japan; ^26^ Clinical Trial Service Unit, Nuffield Department of Population Health Big Data Institute, University of Oxford Oxford United Kingdom; ^27^ Epidemiological Studies Unit, Nuffield Department of Population Health Big Data Institute, University of Oxford Oxford United Kingdom

**Keywords:** prostate cancer risk, phytoestrogens, isoflavones, lignans, pooled analysis

## Abstract

Phytoestrogens may influence prostate cancer development. This study aimed to examine the association between prediagnostic circulating concentrations of isoflavones (genistein, daidzein, equol) and lignans (enterolactone and enterodiol) and the risk of prostate cancer. Individual participant data were available from seven prospective studies (two studies from Japan with 241 cases and 503 controls and five studies from Europe with 2,828 cases and 5,593 controls). Because of the large difference in circulating isoflavone concentrations between Japan and Europe, analyses of the associations of isoflavone concentrations and prostate cancer risk were evaluated separately. Prostate cancer risk by study‐specific fourths of circulating concentrations of each phytoestrogen was estimated using multivariable‐adjusted conditional logistic regression. In men from Japan, those with high compared to low circulating equol concentrations had a lower risk of prostate cancer (multivariable‐adjusted OR for upper quartile [Q4] *vs*. Q1 = 0.61, 95% confidence interval [CI] = 0.39–0.97), although there was no significant trend (OR per 75 percentile increase = 0.69, 95 CI = 0.46–1.05, *p*_trend_ = 0.085); Genistein and daidzein concentrations were not significantly associated with risk (ORs for Q4 *vs*. Q1 = 0.70, 0.45–1.10 and 0.71, 0.45–1.12, respectively). In men from Europe, circulating concentrations of genistein, daidzein and equol were not associated with risk. Circulating lignan concentrations were not associated with the risk of prostate cancer, overall or by disease aggressiveness or time to diagnosis. There was no strong evidence that prediagnostic circulating concentrations of isoflavones or lignans are associated with prostate cancer risk, although further research is warranted in populations where isoflavone intakes are high.

## Abstract 2

**What's new?**

The role of phytoestrogens in prostate cancer development is uncertain. Here, the authors analysed participant data from seven prospective studies on the association between pre‐diagnostic circulating concentrations of isoflavones (mainly found in soybeans) and lignans (mainly found in cereal, nuts, and vegetables) and prostate cancer risk. They found no strong associations but point to the fact that further data are needed to examine associations based on disease aggressiveness, especially in populations with high isoflavone intakes.

**Abbreviations**
BMI: body mass index
CI: confidence interval
OR: odds ratio

## 1 Introduction

It has been hypothesised that a high dietary phytoestrogen intake may inhibit prostate cancer development.^1^, ^2^ Evidence from *in vitro* studies, animal models and observational studies has suggested that this may be due to their effects on hormone metabolism and receptor signalling, or to other mechanisms such as effects on DNA repair, angiogenesis and cell proliferation.^3^. The main types of dietary phytoestrogens are isoflavones and lignans. Soybeans and soya products are the predominant dietary source of isoflavones,^4^ while precursors of mammalian lignans are mainly present in cereals, nuts, seeds and fruits and vegetables.^5^, ^6^ The absorption of phytoestrogens is influenced by the gut microbiota,^7^, ^8^ therefore it is important to measure these compounds in biological samples such as blood (rather than relying solely on an estimation of dietary intake) in order to assess the possible protective effect of phytoestrogens on prostate cancer development.

The Endogenous Hormones, Nutritional Biomarkers and Prostate Cancer Collaborative Group (EHNBPCCG) was established to conduct collaborative analyses of individual participant data from prospective studies on the associations of circulating concentrations of hormones and nutritional biomarkers with risk of prostate cancer.^9^, ^10^, ^11^, ^12^, ^13^, ^14^ The objective of the present study was to examine the associations between concentrations of circulating isoflavones (genistein, daidzein and equol) and lignans (enterolactone and enterodiol), and risk of prostate cancer in a pooled analysis of the worldwide prospective studies.

## 2 Material and Methods

### 2.1 Identification of studies

Studies were eligible to join this collaborative analysis if they had data on circulating phytoestrogens measured in blood samples collected prior to a prostate cancer diagnosis. Studies were identified by using the search terms *phyto‐oestrogens*, *phytoestrogens*, *isoflavones*, *genistein*, *daidzein*, *equol*, *enterolactone* and *enterodiol* together with the MeSH term *prostatic neoplasms* and text term *prostate cancer*. The literature searches were conducted using articles with publication dates through to November 2017 from PubMed, Web of Science, Cochrane Library, CancerLit (up to January 2013) and Google Scholar and from discussions with colleagues. This collaborative group also includes unpublished data obtained from discussion with colleagues, which reduces the risk of publication bias.

### 2.2 Collection and synthesis of data

Individual participant data were available from seven nested case–control studies by the date of dataset closure (November 2017): the European Prospective Investigation into Cancer and Nutrition (EPIC) (cases diagnosed from June 1999 to January 2003 (designated Phase 1);^15^ cases diagnosed from February 2003 to December 2006 (designated Phase 2),^16^ EPIC‐Norfolk^17^ (for participants not included in either the EPIC Phase 1 or Phase 2), Janus Nordic Biological Specimen Biobank Working Group (NBSBWG),^18^, ^19^, ^20^ Japan Collaborative Cohort Study (JACC),^21^ Japan Public Health Center‐based prospective Study (JPHC),^22^ the Malmö Diet and Cancer Study (MDCS)^23^ and the Northern Sweden Health and Disease Cohort (NSHDC).^24^ Three of these studies contributed data on genistein (1,846 cases, 2,200 controls), four had data on daidzein (1,239 cases, 1,675 controls) and equol (1,209 cases, 1,571 controls), five had data on enterolactone (2,828 cases, 5,593 controls) and two studies had data on enterodiol (1,002 cases, 1,197 controls). Data on enterolactone from two studies (the α‐Tocopherol, β‐Carotene Cancer Prevention Study and the Helsinki Heart Study), with a combined total of 350 cases, were not available for pooling.^18^, ^25^

This large collaborative analysis has brought together and reanalysed almost all of the available prospective data on the associations of circulating isoflavone and lignan concentrations with prostate cancer incidence, representing, as far as we are aware, all of the worldwide prospective data on circulating genistein, daidzein and equol, and over 90% of the worldwide data on circulating lignans. While data on enterolactone were not available for this analysis, their results do not differ materially from those reported here, and it is unlikely that these data would have changed our summary risk estimates.

The characteristics of the studies and the assay methods are shown in Tables S1 and S2; detailed information on recruitment, ethics approval and inclusion criteria is available in the original publications.^15^, ^16^, ^17^, ^18^, ^21^, ^22^, ^24^

Where available, collaborators provided data for prostate cancer cases and controls on serum or plasma phytoestrogen concentrations, dates of birth and blood collection, marital status, ethnicity, educational attainment, family history of prostate cancer, height, weight and smoking status. Most studies also provided data on cancer stage and grade and date at diagnosis. Tumour stage was categorised as localised if it was tumour‐node‐metastasis (TNM) stage ≤T2 with no reported lymph node involvement or metastases, stage ≤II, or equivalent; advanced stage if it was T3 or T4 and/or N1+ and/or M1, stage III‐IV, or equivalent; or unknown. Aggressive disease was categorised as “no” for TNM stage ≤T3 with no reported lymph node involvement or metastases or the equivalent, “yes” for TNM stage T4 and/or N1+ and/or M1 and/or stage IV disease or death from prostate cancer, or unknown. Histological grade was categorised as low‐intermediate grade if the Gleason sum was <8, or coded as well, moderately or poorly differentiated; high grade if the Gleason sum was ≥8, or coded as undifferentiated; or grade unknown.

### 2.3 Statistical analyses

The methods of analysis were similar to those described previously by this collaborative group.^10^, ^11^ Because of the large difference in circulating isoflavone concentrations between Asian and Western populations, the analyses for isoflavones were performed separately for Japanese and European studies.

Men were categorised into fourths of concentrations for each phytoestrogen, with cut points defined by the study‐specific quartiles of the distribution in control participants. This approach allows for any systematic differences between the studies, i.e., assay methods, blood sample types or storage conditions.^26^ We used logistic regression conditioned on the matching variables within each study to determine odds ratios (ORs) and their corresponding 95% confidence intervals (CIs). To provide a summary measure of the OR (for subgroup analyses) and to calculate a *p*‐value for trend, the categorical variable representing the fourths of phytoestrogen concentration was replaced with a continuous variable that was scored 0, 0.33, 0.67 and 1, respectively. Because the mid‐points of the lowest and highest fourths are the 12.5 and 87.5 percentage points of the study‐specific phytoestrogen concentration, a unit increase in this variable can be taken to represent a 75 percentile increase in phytoestrogen concentration. The effects of additional potential confounders (other than the matching criteria, which were controlled by study design) were examined by additionally adjusting for variables that were found to be associated with prostate cancer risk in this collaborative dataset,^10^, including age at blood collection (exact), body mass index (BMI = <25, 25–27.4, 27.5–29.9, ≥30 kg/m^2^, unknown), height (≤170, 171–175, 176–180, >180 cm, unknown), marital status (married/cohabiting, not married/cohabiting, unknown), educational status (did not graduate from high school/secondary school/college, high school/secondary school/college graduates, university graduates, unknown) and cigarette smoking (never, past, current, unknown).

Heterogeneity in linear trends between studies was tested by comparing the chi‐squared values for models with and without a (study) × (linear trend) interaction term. To identify evidence of heterogeneity in the associations of phytoestrogens with prostate cancer risk according to case‐defined characteristics (i.e., age at diagnosis [<65 *vs*. ≥ 65 years], years between blood collection and diagnosis [<5 *vs*. ≥ 5 years], year of diagnosis [pre‐2000 *vs*. 2000 onwards], stage [localised *vs*. advanced], aggressiveness [no *vs*. yes] and grade [low‐intermediate *vs*. high]), we fitted separate models for each subgroup and assumed independence of the ORs using a method analogous to a meta‐analysis. Data on tumour stage and/or grade were only available for one of the two Japanese studies,^22^ and hence subgroup analyses of the association of isoflavone concentrations by tumour characteristics in these studies were not performed.

Tests for heterogeneity for non‐case‐defined factors (i.e., age at blood collection [<65 *vs*. ≥ 65 years], BMI [<25 *vs*. ≥ 25 kg/m^2^], cigarette smoking [never or past smoker *vs*. current smoker] and alcohol consumption [<10 *vs*. ≥ 10 g/day]) were assessed with chi‐squared tests of interaction between the subgroup and the continuous trend test variable.

All tests of statistical significance were two sided, and statistical significance was set at the 5% level. All statistical analyses were carried out using Stata version 14.1 (Stata Corp., College Station, TX).

## 3 Results

A total of 241 cases and 503 controls from two Japanese prospective studies, and 2,828 cases and 5,593 controls from five European prospective studies were included in this analysis. The mean age at blood collection in cases or controls across the studies ranged from 46.5 to 68.7 years (Table 1).

**Table 1:** Participant characteristics by study and case–control status

Studies	Case–control status	Participants, *n*	Age at recruitment, years	Height,**1** cm	BMI,**1** kg/m^2^	Married or Cohabiting,**1** %	Higher Education,**1** %	Current Smoker,**1** %
**Japanese**								
JACC (22)	Case	40	68.7 (6.3)	160 (6)	22.4 (2.6)	100	9.7	51.4
	Control	101	67.9 (5.7)	159 (7)	22.4 (2.7)	92.3	26.4	37.8
JPHC (23)	Case	201	59.5 (6.4)	162 (6)	23.4 (2.4)	93.5	–	34.3
	Control	402	59.2 (6.6)	162 (6)	23.3 (2.6)	90.5	–	40.8
**European**								
EPIC, Phase 1 (16)	Case	950	59.9 (5.8)	174 (7)	26.6 (3.4)	89.2	26.9	24.3
	Control	1,042	59.6 (5.8)	174 (7)	26.9 (3.6)	88.9	26.4	29.8
EPIC, Phase 2 (17)	Case	655	59.3 (6.9)	173 (7)	26.9 (3.4)	89.5	25.7	21.5
	Control	655	59.3 (6.9)	173 (7)	26.9 (3.6)	90.2	26.4	23.1
EPIC‐Norfolk (18)	Case	48	65.3 (6.7)	172 (8)	26.3 (2.7)	87.0	12.5	10.4
	Control	130	64.7 (6.4)	173 (6)	26.5 (2.9)	90.8	11.5	10.2
Janus NBSBWG (19)	Case	573	46.5 (4.3)	177 (7)	25.4 (3.1)	–	–	60.9
	Control	2,209	46.5 (4.2)	176 (7)	25.1 (3.2)	–	–	62.4
MDCS (24)	Case	990	60.2 (6.6)	177 (7)	26.2 (3.4)	78.5	15.1	22.3
	Control	1,664	60.0 (6.6)	176 (6)	26.3 (3.4)	74.0	13.5	27.1
NSHDC (25)	Case	261	58.0 (4.4)	176 (6)	26.1 (2.8)	87.1	13.2	18.2
	Control	514	58.0 (4.4)	175 (6)	26.6 (3.5)	79.8	12.2	22.1

The cases and controls are nested in prospective studies, and the numbers of cases and controls are based on the number in complete matched sets for genistein (EPIC, JACC and JPHC), daidzein (EPIC‐Norfolk) and enterolactone (Janus NBSBWG, MDCS and NSHDC).

1 Unknown for some participants.

**Abbreviations:** BMI, body mass index; EPIC, European Prospective Investigation into Cancer and Nutrition; NBSBWG, Janus Nordic Biological Specimen Biobank Working Group; JACC, Japan Collaborative Cohort Study; JPHC, Japan Public Health Center‐based prospective Study; MDCS, Malmö Diet and Cancer Study; NSHDC, Northern Sweden Health and Disease Cohort.

Most of the cases were between 60 and 69 years at diagnosis, except for those from Japanese studies and EPIC‐Norfolk where most cases were diagnosed over 70 years (Table 2). The majority of cases with information on stage and grade had localised disease (ranging from 70 to 87% across studies) and low‐intermediate grade tumours (75 to 98% of cases).

**Table 2:** Characteristics of men who developed prostate cancer and tumour characteristics by study

	Age at diagnosis, %			Date of diagnosis, %		Years from blood collection to diagnosis, %			Disease stage, aggressiveness and grade, %					
Studies	<60	60–69	≥70	Pre‐2000	2000‐	<3	3–6	≥7	Advanced stage**1**	Unknown stage	Aggressive disease**2**	Unknown aggressiveness	High grade**3**	Unknown grade
**Japanese**														
JACC (22)	0	35.0	65.0	100	0	17.5	52.5	30.0	–	100	–	100	–	100
JPHC (23)	7.0	39.8	53.2	25.4	74.6	9.0	17.4	73.6	25.6	24.9	29.9	23.4	24.2	69.1
**European**														
EPIC, Phase 1 (16)	19.4	65.0	15.6	46.7	53.3	31.0	55.7	13.3	28.9	27.2	31.5	21.6	13.6	22.6
EPIC, Phase 2 (17)	14.2	55.0	30.8	3.8	98.2	3.0	42.9	54.0	24.8	28.5	19.5	26.4	11.6	25.0
EPIC‐Norfolk (18)	4.2	33.3	62.5	2.1	97.9	2.1	62.5	35.4	17.8	6.2	31.1	6.2	25.0	91.7
Janus NBSBWG (19)	20.9	69.1	9.9	100	0	1.2	5.1	93.7	–	100	–	100	–	100
MDCS (24)	5.3	48.7	46.1	25.1	74.9	9.5	25.3	65.3	–	100	100^4^	89.7	–	100
NSHDC (25)	16.9	78.5	4.6	72.0	28.0	27.6	49.8	22.6	18.5	0.8	12.7	0.8	1.5	74.3

The percentages are based on the number in complete matched sets for genistein (EPIC, JACC and JPHC), daidzein (EPIC‐Norfolk), and enterolactone (Janus NBSBWG, MDCS and NSHDC).

1 As a percentage of those with known disease stage. Stage of disease was defined as being advanced if it was tumour‐node‐metastasis (TNM) stage T3 or T4 and/or N1+ and/or M1, stages III–IV, or approximate equivalent (that is, a tumour extending beyond the prostate capsule and/or lymph node involvement and/or distant metastases), localised if it was TNM stage T0 or T1 or T2 with no reported lymph node involvement or metastases, stages 0–II, or approximate equivalent (that is, a tumour that does not extend beyond the prostate capsule) or stage unknown.

2 As a percentage of those with known aggressive disease. Aggressive disease was categorised as “yes” for TNM stage T4 and/or N1+ and/or M1 and/or stage IV disease or death from prostate cancer, “no” for TNM stage T0, T1, T2 or T3 with no reported lymph node involvement or metastases or the equivalent, or unknown.

3 As a percentage of those with known disease grade. Grade of disease defined as high grade if the Gleason sum was at least 8 or approximate equivalent (that is, extent of differentiation of “none”), low grade if the Gleason sum was less than 8 or approximate equivalent (that is, extent of differentiation of “poor,” “moderate” or “good”) or grade unknown.

4 Stage and grade of disease are unknown in MDCS, so aggressive disease is defined as cases who died of prostate cancer, but we cannot determine whether cases who *did not die of prostate cancer* had aggressive disease. Therefore, where aggressive disease status is known for this study, aggressive disease status is ‘yes’ and cannot be ‘no.’ **Abbreviations:** EPIC, European Prospective Investigation into Cancer and Nutrition; NBSBWG, Janus Nordic Biological Specimen Biobank Working Group; JACC, Japan Collaborative Cohort Study; JPHC, Japan Public Health Center‐based prospective Study; MDCS, Malmö Diet and Cancer Study; NSHDC, Northern Sweden Health and Disease Cohort.

The geometric means and interquartile ranges of the five phytoestrogens by case–control status are shown in Table 3. There was a large difference in circulating isoflavone concentrations between Japanese and European populations; for example, the mean genistein concentration in controls was between 294.0 and 454.4 nmol/L in Japanese studies and between 5.19 and 5.61 nmol/L in European studies. There was no large variation in circulating lignan concentrations between studies.

**Table 3:** Prediagnostic geometric mean (95% CI) phytoestrogen concentrations (nmol/L) by study in cases and controls

					Lignans	
Studies		Isoflavones Genistein	Daidzein	Equol	Enterolactone	Enterodiol
**Japanese**						
JACC (22)	Case	331.8 (250.6–439.2)	127.6 (92.8–175.3)	10.3 (5.6–19.1)	–	–
	Control	454.4 (361.3–571.6)	166.5 (130.5–212.3)	24.0 (16.2–35.7)	–	–
JPHC (23)	Case	277.2 (235.5–326.2)	115.9 (96.6–139.0)	13.6 (10.6–17.4)	–	–
	Control	294.0 (261.7–330.2)	122.8 (107.7–140.0)	17.4 (14.5–20.9)	–	–
**European**						
EPIC, Phase 1 (16)	Case	4.84 (4.29–5.48)	3.80 (3.42–4.22)	0.65 (0.61–0.70)	11.0 (10.1–11.9)	0.99 (0.91–1.07)
	Control	5.61 (5.00–6.29)	3.96 (3.58–4.38)	0.65 (0.60–0.69)	11.5 (10.6–12.4)	0.99 (0.92–1.08)
EPIC, Phase 2 (17)	Case	5.97 (5.26–6.79)	–	–	–	–
	Control	5.19 (4.59–5.87)	–	–	–	–
EPIC‐Norfolk (18)	Case	–	3.31 (2.08–5.27)	0.59 (0.28–1.24)	4.98 (3.74–6.63)	0.23 (0.14–0.36)
	Control	–	2.84 (2.28–3.54)	0.25 (0.14–0.44)	4.89 (4.10–5.83)	0.18 (0.15–0.22)
Janus NBSBWG (19)	Case	–	–	–	6.55 (6.10–7.03)	–
	Control	–	–	–	5.82 (5.58–6.07)	–
MDCS (24)	Case	–	–	–	9.57 (8.90–10.3)	–
	Control	–	–	–	9.58 (9.06–10.1)	–
NSHDC (25)	Case	–	–	–	15.5 (13.6–17.6)	–
	Control	–	–	–	14.6 (13.4–15.9)	–

**Abbreviations**: EPIC, European Prospective Investigation into Cancer and Nutrition; NBSBWG, Janus Nordic Biological Specimen Biobank Working Group; JACC, Japan Collaborative Cohort Study; JPHC, Japan Public Health Center‐based prospective Study; MDCS, Malmö Diet and Cancer Study; NSHDC, Northern Sweden Health and Disease Cohort.

**Figure 1:**
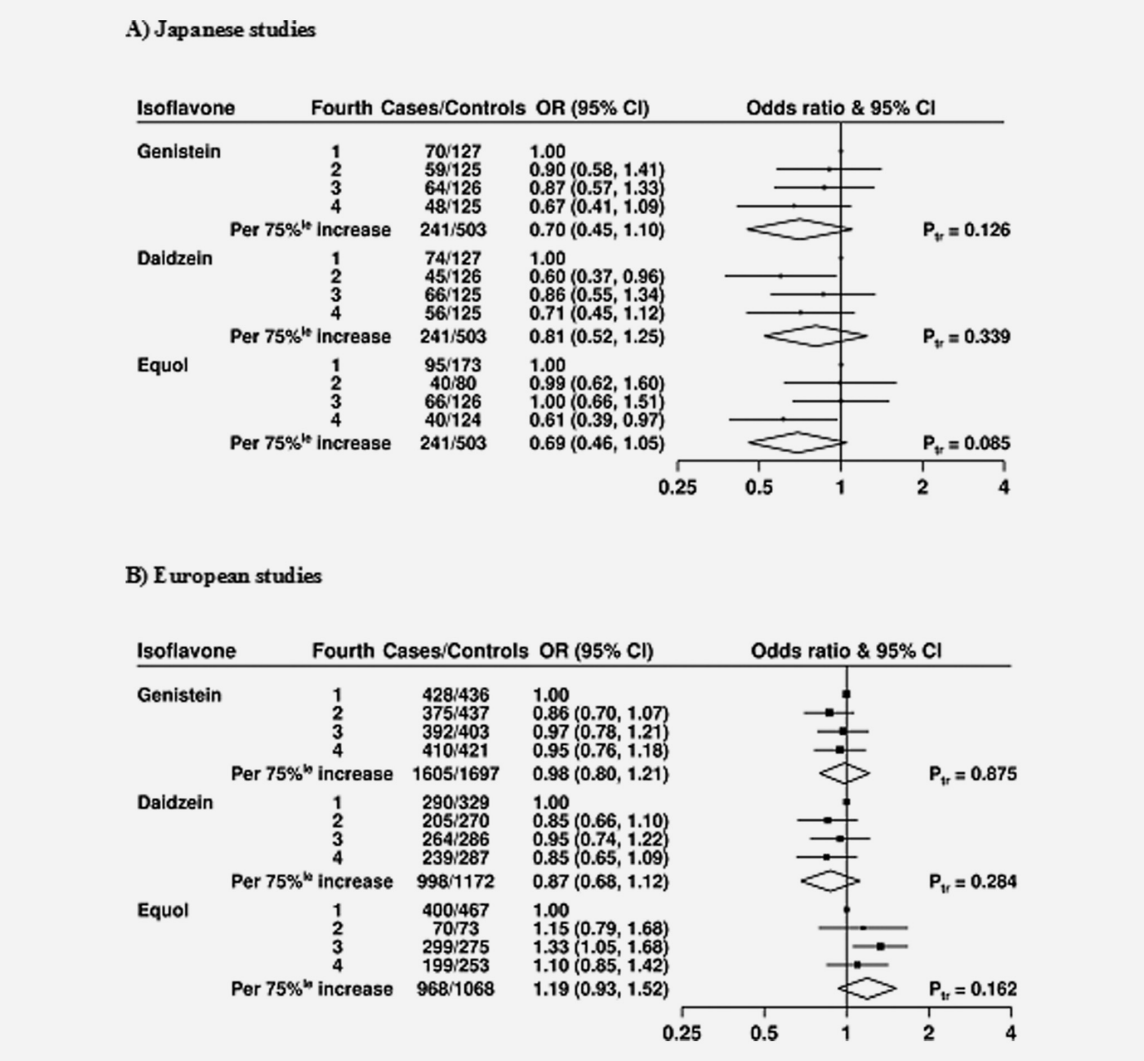
ORs for prostate cancer associated with isoflavone concentrations in Japanese (*a*) and European (*b*) studies. The black squares indicate the ORs in study‐specific fourths, and the horizontal lines show the 95% CIs. The area of each square is proportional to the amount of statistical information (inverse of the variance of the logarithm of the OR). The diamonds show the OR for an increase in concentration from the 12.5 and 87.5 percentage points, and the widths of the diamonds show the 95% CIs. The *χ*2 tests for linear trend (Ptr) were calculated scoring the fourths as 0, 0.33, 0.67 and 1. Estimates are from conditional logistic regression on case‐control sets matched within each study and adjusted for age at blood collection (exact), body mass index (BMI; <25, 25–27.4, 27.5–29.9 and ≥30 kg/m^2^, unknown), height (≤170, 171–175, 176–180 and >180 cm, unknown), marital status (married/cohabiting, not married/cohabiting, unknown), educational status (did not graduate from high school/secondary school/college, high school/secondary school/college graduates, university graduates, unknown) and cigarette smoking (never, past, current, unknown).

The ORs for prostate cancer risk by fourths of circulating isoflavone concentrations (separately by Japanese and European studies), adjusted for age, marital status, educational attainment, smoking, height and BMI are shown in **Figure** 1. In men from Japan, there was some evidence of an inverse association between circulating equol and prostate cancer risk (multivariable‐adjusted OR for the highest versus lowest fourth = 0.61, 95% confidence interval [CI] = 0.39–0.97), although there was no significant trend (OR per 75 percentile increase = 0.69, 95 CI = 0.46–1.05, *p*_trend_ = 0.085). Genistein and daidzein concentrations were not significantly associated with risk (ORs for the highest versus lowest fourth = 0.70, 0.45–1.10 and 0.71, 0.45–1.12, respectively). In men from Europe, circulating concentrations of genistein, daidzein and equol were not associated with risk.

**Figure 2:**
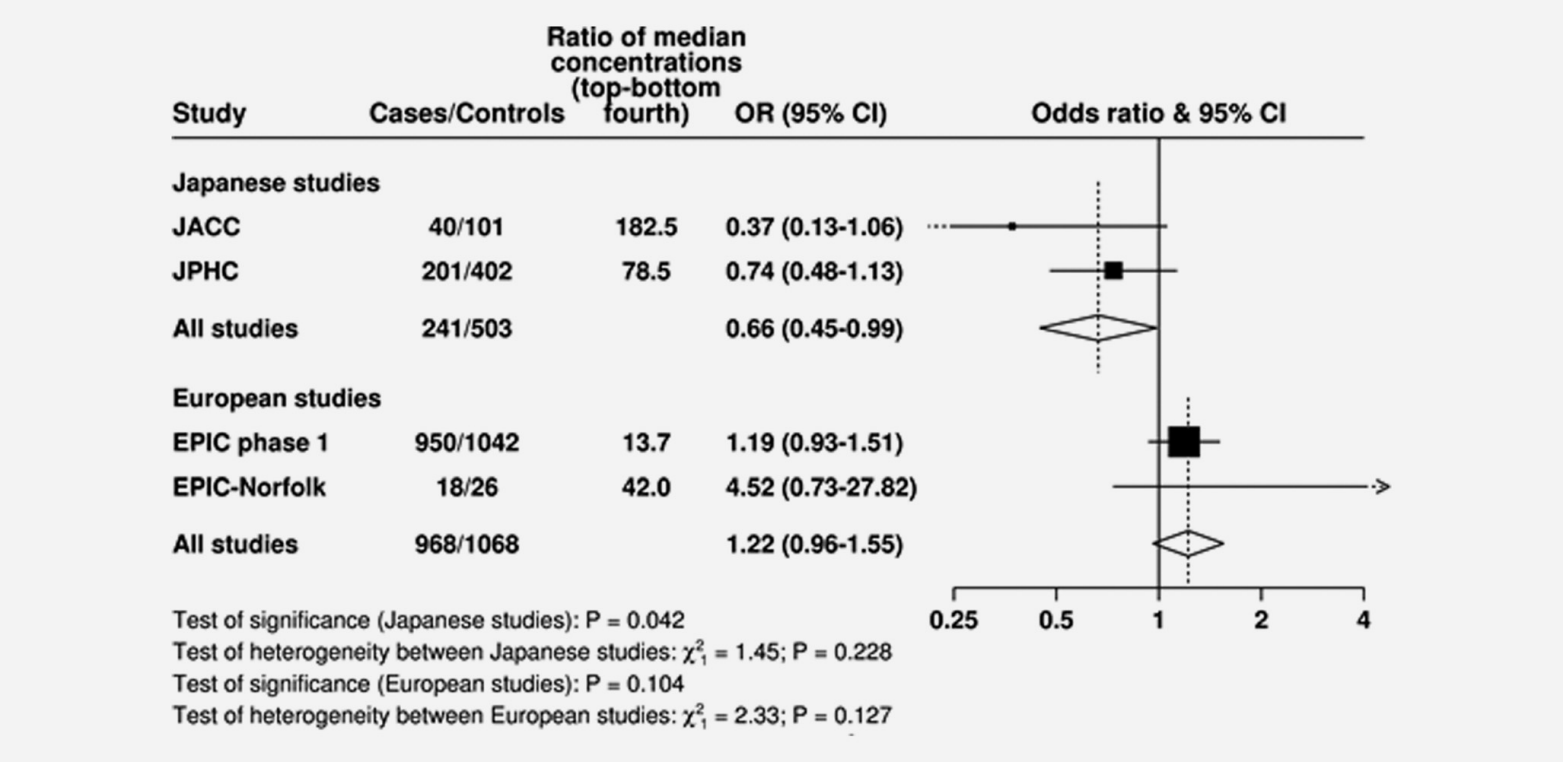
Study‐specific ORs (95% CIs) for prostate cancer associated with a 75 percentile increase in equol concentrations. Estimates are from logistic regression conditioned on the matching variables within each study, but not further adjusted. Heterogeneity in linear trends between studies and between Japanese and European studies was tested by comparing the *χ*2 values for models with and without a (studies) × (linear trend) interaction term. Abbreviations: European Prospective Investigation into Cancer and Nutrition (EPIC), Japan Collaborative Cohort Study (JACC), Japan Public Health Center‐based prospective Study (JPHC).

**Figure** 2 and **Figures** S1 **and** S2 show the associations of isoflavones with prostate cancer risk for each study. Note that because of the small number of cases in some studies and missing data on some covariates, these analyses were not adjusted for anthropometric and lifestyle factors; therefore, the results shown in these figures for all studies combined differ slightly from those shown in Figure 1. For genistein, there was evidence of heterogeneity within European studies (*p*_heterogeneity_ = 0.001): the OR for a 75 percentile increase in genistein was 0.73 (95% CI: 0.56–0.95) in EPIC Phase 1, while it was 1.51 (95% CI: 1.06–2.13) in EPIC phase 2 (**Fig.** S1).

The associations of isoflavone concentrations with prostate cancer risk subdivided by time to diagnosis and other characteristics are shown in **Figures** S3–S8. In Japanese studies, the associations of genistein and equol concentrations with prostate cancer risk did not differ significantly by age at diagnosis, time to diagnosis, year of diagnosis, age at blood collection, smoking status or alcohol consumption (**Figs.** S3 **and** S5). For daidzein, there was significant heterogeneity by year of diagnosis (*p*_heterogeneity_ = 0.032) with a reduction in risk of prostate cancer for cases diagnosed before the year 2000 (OR per 75 percentile increase = 0.43, 95% CI = 0.20–0.92) but not in cases diagnosed later (OR per 75 percentile increase = 1.21, 95% CI = 0.69–2.12) (**Fig.** S4).

In European studies, we found no evidence of heterogeneity for the association of isoflavones with prostate cancer according to time to diagnosis or other characteristics (**Figs.** S6–S8), with the exception of daidzein and BMI (*p*_heterogeneity_ = 0.028). Here, daidzein was associated with a reduced risk of prostate cancer in men with a BMI ≥ 25 kg/m^2^ (OR per 75 percentile increase = 0.72, 95% CI = 0.52–0.98) but not in normal‐weight men (OR per 75 percentile increase = 1.29, 95% CI = 0.84–2.00) (**Fig.** S7).

**Figure 3:**
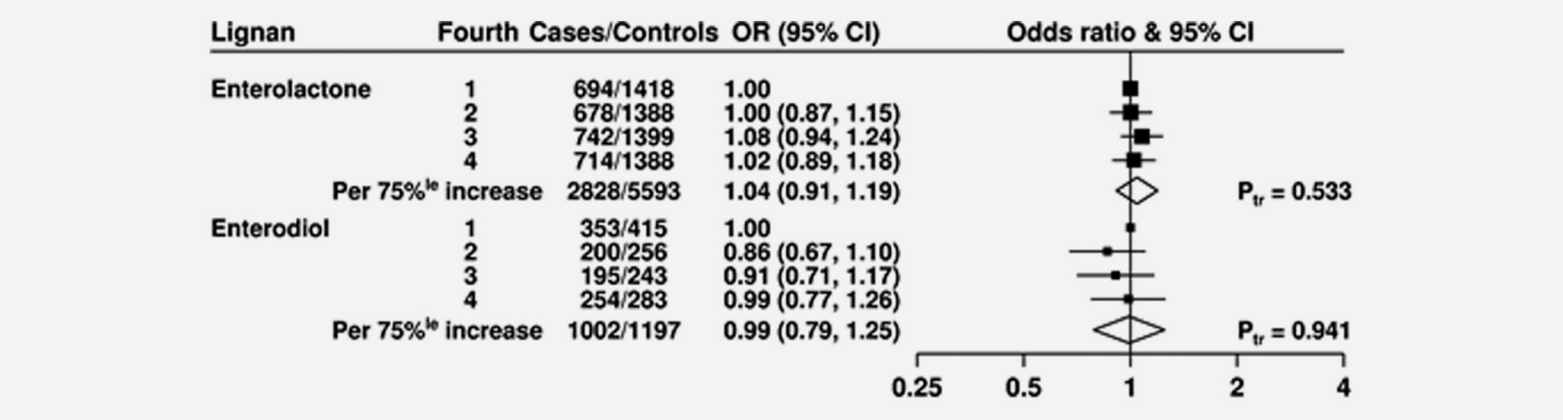
ORs for prostate cancer associated with lignan concentrations. The black squares indicate the ORs in study‐specific fourths, and the horizontal lines show the 95% CIs. The area of each square is proportional to the amount of statistical information (inverse of the variance of the logarithm of the OR). The diamonds show the OR for an increase in concentration from the 12.5 and 87.5 percentage points, and the widths of the diamonds show the 95% CIs. The *χ*2 tests for linear trend (Ptr) were calculated scoring the fourths as 0, 0.33, 0.67 and 1. Estimates are from conditional logistic regression on case‐control sets matched within each study and adjusted for age at blood collection (exact), body mass index (BMI; <25, 25–27.4, 27.5–29.9 and ≥30 kg/m^2^, unknown), height (≤170, 171–175, 176–180 and >180 cm, unknown), marital status (married/cohabiting, not married/cohabiting and unknown), educational status (did not graduate from high school/secondary school/college, high school/secondary school/college graduates, university graduates, unknown) and cigarette smoking (never, past, current and unknown).

No associations between circulating lignan concentrations and prostate cancer risk were observed (OR per 75 percentile increase = 1.04, 95 CI = 0.91–1.19 for enterolactone and OR per 75 percentile increase = 0.99, 95 CI = 0.79–1.25 for enterodiol) (**Fig**. 3 **)**. There was also no evidence of heterogeneity in these associations between the different studies (**Figs.** S9 **and** S10) and these associations did not differ by tumour characteristics or lifestyle factors (**Figs.** S11 **and** S12).

## 4 Discussion

The results of this collaborative analysis of individual participant data show no strong evidence that prediagnostic circulating concentrations of isoflavones or lignans are associated with prostate cancer risk. Equol concentrations were weakly inversely related with overall prostate cancer risk in Japanese populations; this association was significant when we compared the highest with the lowest fourth, but we did not find a significant linear trend. When we looked into subtypes, we found some evidence of heterogeneity in three subgroups; however, given the number of comparisons made in our study, these may be chance findings.

This large collaborative analysis has brought together and reanalysed almost all of the available prospective data on the associations of circulating isoflavone and lignan concentrations with prostate cancer incidence, representing, as far as we are aware, all of the worldwide prospective data on circulating genistein, daidzein and equol, and over 90% of the worldwide data on circulating lignans. While data on enterolactone from the α‐Tocopherol, β‐Carotene Cancer Prevention Study (214 case patients)^25^ and the Helsinki Heart Study (136 case patients)^18^ were not available for this analysis, their results do not differ materially from those reported here, and it is unlikely that these data would have changed our summary risk estimates. The Multiethnic Cohort (MEC) study,^27^ in which phytoestrogen concentrations were measured in urine, found a weak inverse association between genistein and daidzein concentrations and prostate cancer risk, which did not differ across the four ethnic groups examined (African Americans, Japanese Americans, Latinos, Whites). However, this study was not included in these analyses because urine concentrations are not comparable to circulating concentrations, and because there was no other study with urinary phytoestrogen measures with which to pool these data.

Some studies have studies whether prostate cancer risk is related to the dietary intake of soya foods, but a meta‐analysis of prospective studies reported no association.^28^ A meta‐analysis of randomised controlled trials on the effect of soya supplementation (mainly combining genistein, daidzein and glycitein) on prostate cancer risk found only two small trials in which the endpoint included precursor lesions.^29^ Thus, conclusions on prostate cancer prevention cannot be drawn.

Equol is a metabolite produced in the gastrointestinal tract by bacterial metabolism of daidzein, and there is some evidence that equol has higher hormonal activity that genistein and daidzein.^30^, ^31^ *In vitro* and animal studies have shown that equol has anti‐androgenic and anti‐proliferative properties in the prostate,^30^, ^32^ but its role in humans remains unclear. Equol production varies between individuals, and the term equol‐producers refers to people who produce equol after consuming isoflavones; therefore it may be possible that the health benefits of soya‐based diets are greater in equol‐producers than in equol non‐producers.^33^. Only between 20 and 50% of individuals can produce equol,^34^, ^35^ and the capacity to produce equol has been found to be lower among American than Asian men,^36^ perhaps because Asian populations consume dietary isoflavones from an early age.

Both enterolactone and enterodiol are formed from plant‐lignan glycoside precursors by the activity of the gut microbiota in the proximal colon.^31^ Both *in vitro* and clinical studies have suggested that a diet rich in precursors of mammalian lignans (e.g., whole grain rye, bran or flaxseed) may have antiproliferative effects in the early stages of prostate cancer development.^37^, ^38^, ^39^ However, findings from the current study do not support the hypothesis that circulating lignans play a role in the aetiology of prostate cancer.

The current analyses have several strengths and limitations. Strengths include the large sample size, the detailed data on participant characteristics, and the consistent statistical approach used to analyse the individual participant data across the studies. Moreover, this collaboration only includes prospective studies, which allowed us to assess whether associations varied by time from blood collection to diagnosis and hence examine possible reverse causality. The limitations of these analyses include the use of a single blood sample measurement of phytoestrogens, as the true exposure of interest is medium to long‐term average levels of circulating phytoestrogens. Two studies in women have shown that the within‐person reproducibility (over a 1‐ to 3‐year period) is relatively poor for daidzein and genistein.^40^, ^41^ To our knowledge, only one such reproducibility study has been carried out in men, which showed an intra‐class correlation coefficient (ICC) for plasma genistein of 0.32 (95% CI 0.14–0.50) for samples collected approximately 5 years apart.^16^ However, these studies were performed in Western populations, where the consumption of isoflavones is usually low and episodic over time; therefore a single measurement of circulating isoflavones is unlikely to represent long‐term average exposure and may have led to attenuation of risk estimates. Low to moderate ICCs over several years (over a 1‐ to 3‐year period) have also been found for circulating enterolactone (ICCs of 0.52^40^ and 0.55^41^) and enterodiol (ICCs of 0.37^41^) in women. Although we have included almost all of the worldwide prospective data on circulating phytoestrogens and prostate cancer, we had limited power to assess associations by disease aggressiveness and other important sub‐groups, especially in Japanese populations, where isoflavone intake is high. Our study did not have sufficient mortality data to perform separate analyses using prostate cancer death as the outcome, but a recent cohort study found no association between prediagnostic enterolactone concentrations and mortality among men diagnosed with prostate cancer.^42^.

## 5 Conclusion

There was no strong evidence that prediagnostic circulating concentrations of isoflavones or lignans are associated with prostate cancer risk. However, further data are needed to examine these associations by disease aggressiveness, especially in populations following traditional East Asian diets.

## Supporting information


**Supplementary Table 1**. Study characteristics, including sample population, recruitment and assessment characteristics.
**Supplementary Table 2**. Assay details
**Supplementary Figure 1**. Study‐specific ORs (95% CIs) for prostate cancer associated with a 75 percentile increase in genistein concentrations. Estimates are from logistic regression conditioned on the matching variables within each study, but not further adjusted. Heterogeneity in linear trends between studies and between Japanese and European studies was tested by comparing the χ^2^ values for models with and without a (studies) x (linear trend) interaction term. Abbreviations: European Prospective Investigation into Cancer and Nutrition (EPIC), Japan Collaborative Cohort Study (JACC), Japan Public Health Center‐based prospective Study (JPHC).
**Supplementary Figure 2**. Study‐specific ORs (95% CIs) for prostate cancer associated with a 75 percentile increase in daidzein concentrations. Estimates are from logistic regression conditioned on the matching variables within each study, but not further adjusted. Heterogeneity in linear trends between studies and between Japanese and European studies was tested by comparing the χ^2^ values for models with and without a (studies) x (linear trend) interaction term. Abbreviations: European Prospective Investigation into Cancer and Nutrition (EPIC), Japan Collaborative Cohort Study (JACC), Japan Public Health Center‐based prospective Study (JPHC).
**Supplementary Figure 3**. ORs for prostate cancer associated with genistein concentration, according to characteristics of cases and controls in Japanese studies. Each OR is the estimate of the linear trend obtained by replacing the categorical variables representing the fourths of genistein concentration by a continuous variable scored as 0, 0.33, 0.67, and 1. Black squares indicate the OR, and the horizontal lines show the 95% CIs. The area of each square is proportional to the amount of statistical information (inverse of the variance of the logarithm of the OR). The vertical dotted line indicates the OR for all studies. Tests for heterogeneity are for the difference in the association of genistein with prostate cancer risk between subgroups. Estimates are from conditional logistic regression on case‐control sets matched within each study and adjusted for age at blood collection (exact), body mass index (BMI; <25, 25–27.4, 27.5–29.9, ≥30 kg/m^2^, unknown), height (≤170, 171–175, 176–180, >180 cm, unknown), marital status (married/cohabiting, not married/cohabiting, unknown), and cigarette smoking (never, past, current, unknown).
**Supplementary Figure 4**. ORs for prostate cancer associated with daidzein concentration, according to characteristics of cases and controls in Japanese studies. Each OR is the estimate of the linear trend obtained by replacing the categorical variables representing the fourths of daidzein concentration by a continuous variable scored as 0, 0.33, 0.67, and 1. Black squares indicate the OR, and the horizontal lines show the 95% CIs. The area of each square is proportional to the amount of statistical information (inverse of the variance of the logarithm of the OR). The vertical dotted line indicates the OR for all studies. Tests for heterogeneity are for the difference in the association of daidzein with prostate cancer risk between subgroups. Estimates are from conditional logistic regression on case‐control sets matched within each study and adjusted for age at blood collection (exact), body mass index (BMI; <25, 25–27.4, 27.5–29.9, ≥30 kg/m^2^, unknown), height (≤170, 171–175, 176–180, >180 cm, unknown), marital status (married/cohabiting, not married/cohabiting, unknown), and cigarette smoking (never, past, current, unknown).
**Supplementary Figure 5**. ORs for prostate cancer associated with equol concentration, according to characteristics of cases and controls in Japanese studies. Each OR is the estimate of the linear trend obtained by replacing the categorical variables representing the fourths of equol concentration by a continuous variable scored as 0, 0.33, 0.67, and 1. Black squares indicate the OR, and the horizontal lines show the 95% CIs. The area of each square is proportional to the amount of statistical information (inverse of the variance of the logarithm of the OR). The vertical dotted line indicates the OR for all studies. Tests for heterogeneity are for the difference in the association of equol with prostate cancer risk between subgroups. Estimates are from conditional logistic regression on case‐control sets matched within each study and adjusted for age at blood collection (exact), body mass index (BMI; <25, 25–27.4, 27.5–29.9, ≥30 kg/m^2^, unknown), height (≤170, 171–175, 176–180, >180 cm, unknown), marital status (married/cohabiting, not married/cohabiting, unknown), and cigarette smoking (never, past, current, unknown).
**Supplementary Figure 6**. ORs for prostate cancer associated with genistein concentration, according to characteristics of cases and controls in European studies. Each OR is the estimate of the linear trend obtained by replacing the categorical variables representing the fourths of genistein concentration by a continuous variable scored as 0, 0.33, 0.67, and 1. Black squares indicate the OR, and the horizontal lines show the 95% CIs. The area of each square is proportional to the amount of statistical information (inverse of the variance of the logarithm of the OR). The vertical dotted line indicates the OR for all studies. Tests for heterogeneity are for the difference in the association of genistein with prostate cancer risk between subgroups. Estimates are from conditional logistic regression on case‐control sets matched within each study and adjusted for age at blood collection (exact), body mass index (BMI; <25, 25–27.4, 27.5–29.9, ≥30 kg/m^2^, unknown), height (≤170, 171–175, 176–180, >180 cm, unknown), marital status (married/cohabiting, not married/cohabiting, unknown), educational status (did not graduate from high school/secondary school/college, high school/secondary school/college graduates, university graduates, unknown), and cigarette smoking (never, past, current, unknown).
**Supplementary Figure 7**. ORs for prostate cancer associated with daidzein concentration, according to characteristics of cases and controls in European studies. Each OR is the estimate of the linear trend obtained by replacing the categorical variables representing the fourths of daidzein concentration by a continuous variable scored as 0, 0.33, 0.67, and 1. Black squares indicate the OR, and the horizontal lines show the 95% CIs. The area of each square is proportional to the amount of statistical information (inverse of the variance of the logarithm of the OR). The vertical dotted line indicates the OR for all studies. Tests for heterogeneity are for the difference in the association of daidzein with prostate cancer risk between subgroups. Estimates are from conditional logistic regression on case‐control sets matched within each study and adjusted for age at blood collection (exact), body mass index (BMI = <25, 25–27.4, 27.5–29.9, ≥30 kg/m^2^, unknown), height (≤170, 171–175, 176–180, >180 cm, unknown), marital status (married/cohabiting, not married/cohabiting, unknown), educational status (did not graduate from high school/secondary school/college, high school/secondary school/college graduates, university graduates, unknown), and cigarette smoking (never, past, current, unknown).
**Supplementary Figure 8**. ORs for prostate cancer associated with equol concentration, according to characteristics of cases and controls in European studies. Each OR is the estimate of the linear trend obtained by replacing the categorical variables representing the fourths of equol concentration by a continuous variable scored as 0, 0.33, 0.67, and 1. Black squares indicate the OR, and the horizontal lines show the 95% CIs. The area of each square is proportional to the amount of statistical information (inverse of the variance of the logarithm of the OR). The vertical dotted line indicates the OR for all studies. Tests for heterogeneity are for the difference in the association of equol with prostate cancer risk between subgroups. Estimates are from conditional logistic regression on case‐control sets matched within each study and adjusted for age at blood collection (exact), body mass index (BMI = <25, 25–27.4, 27.5–29.9, ≥30 kg/m^2^, unknown), height (≤170, 171–175, 176–180, >180 cm, unknown), marital status (married/cohabiting, not married/cohabiting, unknown), educational status (did not graduate from high school/secondary school/college, high school/secondary school/college graduates, university graduates, unknown), and cigarette smoking (never, past, current, unknown).
**Supplementary Figure 9**. Study‐specific ORs (95% CIs) for prostate cancer associated with a 75 percentile increase in enterolactone concentrations. Estimates are from logistic regression conditioned on the matching variables within each study, but not further adjusted. Heterogeneity in linear trends between studies and between Japanese and European studies was tested by comparing the χ^2^ values for models with and without a (studies) x (linear trend) interaction term. Abbreviations: European Prospective Investigation into Cancer and Nutrition (EPIC), Janus Nordic Biological Specimen Biobank Working Group (NBSBWG), the Malmö Diet and Cancer Study (MDCS), Northern Sweden Health and Disease Cohort (NSHDC)
**Supplementary Figure 10**. Study‐specific ORs (95% CIs) for prostate cancer associated with a 75 percentile increase in enterodiol concentrations. Estimates are from logistic regression conditioned on the matching variables within each study, but not further adjusted. Heterogeneity in linear trends between studies and between Japanese and European studies was tested by comparing the χ^2^ values for models with and without a (studies) x (linear trend) interaction term. Abbreviation: European Prospective Investigation into Cancer and Nutrition (EPIC).
**Supplementary Figure 11**. ORs for prostate cancer associated with enterolactone concentration, according to characteristics of cases and controls. Each OR is the estimate of the linear trend obtained by replacing the categorical variables representing the fourths of enterolactone concentration by a continuous variable scored as 0, 0.33, 0.67, and 1. Black squares indicate the OR, and the horizontal lines show the 95% CIs. The area of each square is proportional to the amount of statistical information (inverse of the variance of the logarithm of the OR). The vertical dotted line indicates the OR for all studies. Tests for heterogeneity are for the difference in the association of enterolactone with prostate cancer risk between subgroups. Estimates are from conditional logistic regression on case‐control sets matched within each study, but not further adjusted.
**Supplementary Figure 12**. ORs for prostate cancer associated with enterodiol concentration, according to characteristics of cases and controls. Each OR is the estimate of the linear trend obtained by replacing the categorical variables representing the fourths of enterodiol concentration by a continuous variable scored as 0, 0.33, 0.67, and 1. Black squares indicate the OR, and the horizontal lines show the 95% CIs. The area of each square is proportional to the amount of statistical information (inverse of the variance of the logarithm of the OR). The vertical dotted line indicates the OR for all studies. Tests for heterogeneity are for the difference in the association of enterodiol with prostate cancer risk between subgroups. Estimates are from conditional logistic regression on case‐control sets matched within each study, but not further adjusted.
