# Supplementary material for: Circulating isoflavone and lignan concentrations and prostate cancer risk: a meta‐analysis of individual participant data from seven prospective studies including 2,828 cases and 5,593 controls
Source: Int J Cancer. 2018 Sep 29;143(11):2677–86. doi: 10.1002/ijc.31640 (PMC6283047; doi:10.1002/ijc.31640)
Supplement: Supplementary file 1 — Supplementary Table 1. Study characteristics, including sample population, recruitment and assessment characteristics. Supplementary Table 2. Assay details Supplementary Figure 1. Study‐specific ORs (95% CIs) for prostate cancer associated with a 75 percentile increase in genistein concentrations. Estimates are from logistic regression conditioned on the matching variables within each study, but not further adjusted. Heterogeneity in linear trends between studies and between Japanese and European studies was tested by comparing the χ2 values for models with and without a (studies) x (linear trend) interaction term. Abbreviations: European Prospective Investigation into Cancer and Nutrition (EPIC), Japan Collaborative Cohort Study (JACC), Japan Public Health Center‐based prospective Study (JPHC). Supplementary Figure 2. Study‐specific ORs (95% CIs) for prostate cancer associated with a 75 percentile increase in daidzein concentrations. Estimates are from logistic regression conditioned on the matching variables within each study, but not further adjusted. Heterogeneity in linear trends between studies and between Japanese and European studies was tested by comparing the χ2 values for models with and without a (studies) x (linear trend) interaction term. Abbreviations: European Prospective Investigation into Cancer and Nutrition (EPIC), Japan Collaborative Cohort Study (JACC), Japan Public Health Center‐based prospective Study (JPHC). Supplementary Figure 3. ORs for prostate cancer associated with genistein concentration, according to characteristics of cases and controls in Japanese studies. Each OR is the estimate of the linear trend obtained by replacing the categorical variables representing the fourths of genistein concentration by a continuous variable scored as 0, 0.33, 0.67, and 1. Black squares indicate the OR, and the horizontal lines show the 95% CIs. The area of each square is proportional to the amount of statistical information (inverse of the variance o [file IJC-143-2677-s001.docx]

**Funding for the original studies**

Details of funding for the original studies are in the relevant publications. In summary, the funding provided to the collaborating studies was as follows: EPIC: EPIC-Greece: the Hellenic Health Foundation. EPIC-Italy: Associazione Italiana per la Ricerca sul Cancro-AIRC-Italy. MORGEN-EPIC cohort of the EPIC Bilthoven centre: Dutch Ministry of Public Health, Welfare and Sports (VWS), Netherlands Cancer Registry (NKR), Statistics Netherlands (the Netherlands). EPIC-Denmark: Danish Cancer Society. EPIC-Norfolk: Medical Research Council and Cancer Research UK. Janus NBSBWG: The Norwegian Institute of Public Health for access to survey data in this study. The Nordic Cancer Union; Grant sponsor: Phytoprevent Project of the European Commission; Grant sponsor: Sigrid Juselius Foundation, Helsinki, Finland. JACC: Grant-in-Aid for Scientific Research on Priority Areas (2) (No. 14031221) from the Ministry of Education, Culture, Sports, Science and Technology of Japan. The JACC Study has also been supported by Grants-in-Aid for Scientific Research (Nos. 61010076, 62010074, 63010074, 1010068, 2151065, 3151064, 4151063, 5151069, 6279102, and 11181101) from the same Ministry. JPHC: Grants-in-Aid for Cancer Research (Grant No. 19shi-2), for the 3rd Term Comprehensive 10-Year Strategy for Cancer Control (Grant No. H18-sanjigan-ippan-001), and for Research on Risk of Chemical Substances (Grant No. H17-kagakuippan-014) from the Ministry of Health, Labour and Welfare of Japan, and Grants-in-Aid for Scientific Research on Priority Areas from the Ministry of Education, Culture, Sports, Science and Technology (Grant No. 17015049). MDCS: Funding was obtained from the Swedish Council for Working Life and Social Research, Swedish Cancer Society, the Albert Påhlsson Foundation for Scientific Research, the Gunnar Nilsson Cancer Foundation, Skåne University Hospital – Foundations and Donations, the Malmö General Hospital Foundation for the Combating of Cancer, and the Ernhold Lundström Foundation for Scientific Research. NSHDC: Swedish Cancer foundation, Project No. 4620, the European Union, Project No. QRLT 2000-00266. Asa Agren serves as co-ordinator for the Northern Sweden Health and Disease Cohort

| **Supplementary Table 1.** Study characteristics, including sample population, recruitment and assessment characteristics. | | | | | | | | | | | | |
| --- | --- | --- | --- | --- | --- | --- | --- | --- | --- | --- | --- | --- |
| **Study, year (reference)** |  | **Sample population** |  | **Location** |  | **Study recruitment dates** |  | **Prostate cancer ascertainment method** |  | **Nested case-control study characteristics** | | |
|  |  |  |  |  |  |  |  |  |  | **Cases:controls** |  | **Matching criteria and other comments** |
| **Japanese studies** |  |  |  |  |  |  |  |  |  |  |  |  |
| JACC, 2004 (22) |  | Population-based cohort study |  | Japan |  | 1988-1990 |  | Cancer registry linkage (Cancer registries in 24 study areas out of 45) |  | 1:3 |  | Study area and age |
| JPHC, 2008 (23) |  | Population-based cohort study |  | Japan |  | 1990-1995 |  | Active patient notification from major local hospitals in the study area and data linkage with population-based cancer registries, with permission from the local governments responsible for the registries |  | 1:2 |  | Age (within 3 years), Public Health Center area, area (city or town and village), date on which blood was obtained (within 60 days), time of day of blood collection (within 3 hours), and duration of fasting at blood collection |
| **European studies** |  |  |  |  |  |  |  |  |  |  |  |  |
| EPIC, Phase 1 and Phase 2, 2009 and 2012 (16-17) |  | Population-based cohort study |  | Europe |  | 1992-2000 |  | Cancer registry linkage (Denmark, Italy, Netherlands, Spain, Sweden, UK), self-report with medical record review (Germany, Greece) |  | 1:1 except for the Umeå centre which was 1:2 |  | Recruitment center, age at enrolment (±6 months), time of day of blood collection (±1 h), follow-up time (as close as possible), time between blood draw and last consumption of food or drinks (<3, 3–6,>6 h) |
| EPIC-Norfolk, 2008 (18) |  | Population-based cohort study |  | UK |  | 1993-1997 |  | East Anglia Cancer Registry |  | 1:4 |  | Age (± 3 years) |
| Janus NBSBWG, 2002 (19) |  | Population-based cohort study |  | Finland, Norway and Sweden |  | 1981-1991 |  | Cancer registry linkage |  | 1:1 |  | Age (±6 months) and date (±2 months) of the blood sampling |
| MDCS (24) |  | Population-based cohort study |  | Sweden |  |  |  | Swedish Cancer Register and the National Cancer Register |  | 1:2 |  | Date of birth and date of baseline venipuncture within 3 mo of the case who were alive and without a cancer diagnosis at the follow-up time at which the index case was diagnosed |
| NSHDC, 2004 (25) |  | Population-based cohort study |  | Sweden |  | 1985-on going |  | Linkage with the regional cancer registry, using a nation-wide individual identification number as the identity link |  | 1:2 |  | Age (±6 months) and date (±2 months) of the blood sampling |
| Abbreviations: European Prospective Investigation into Cancer and Nutrition (EPIC), Janus Nordic Biological Specimen Biobank Working Group (NBSBWG), Japan Collaborative Cohort Study (JACC), Japan Public Health Center-based prospective Study (JPHC), Malmö Diet and Cancer Study (MDCS), Northern Sweden Health and Disease Cohort (NSHDC), United Kingdom (UK). | | | | | | | | | | | | |

| **Supplementary Table 2.** Assay details | | | | | | | | | | |
| --- | --- | --- | --- | --- | --- | --- | --- | --- | --- | --- |
| **Studies** |  | **Sample** |  | **Method** |  | **Laboratory** |  | **Within-batch CV** |  | **Between batch or overall CV** |
| **Japanese** |  |  |  |  |  |  |  |  |  |  |
| JACC (22) |  | Serum |  | LC/MS/MS |  | SRL, Hachioji, Japan |  | - |  | Total variation of measurements was examined for two samples for quality control in this study; CVs were 6.5% and 7.5% for genistein, 6.9% and 8.2% for daidzein, 8.2% and 9.1% for equol |
| JPHC (23) |  | Plasma |  | Liquid chromatography-mass spectrometry |  | SRL, Tokyo, Japan |  | - |  | Based on 40 replicated measurements of QC samples, interbatch CV were 6.08% for genistein, 4.06% for daidzein, and 6.15% for equol |
| **European** |  |  |  |  |  |  |  |  |  |  |
| EPIC, Phase 1 (16) |  | Plasma |  | Liquid chromatography/tandem mass spectrometry (UPLC-MS/MS) |  | HFL laboratory, Fordham, United Kingdom |  | The average intra-assay CVs ranged from 3.1 (enterolactone) to 5.9% (equol) |  | The average inter-assay CVs ranged from 4.1 (enterodiol) to 6.2% (equol) |
| EPIC, Phase 2 (17) |  | Plasma |  | Liquid chromatography/tandem mass spectrometry (UPLC-MS/MS) |  | HFL laboratory, Fordham, United Kingdom |  | The average intra-assay CV for genistein was 3.6%. |  | The average inter-assay CV for genistein was 7.2 %. |
| EPIC-Norfolk (18) |  | Serum |  | Liquid chromatography/mass spectrometry |  | MRC Dunn Human Nutrition Unit, Cambridge, United Kingdom |  | Intrabatch and interbatch CVs were <4% except equol (5.7%). |  | Intrabatch and interbatch CVs were <4% except equol (5.7%). |
| Janus NBSBWG (19) |  | Serum: The Finnish and Norwegian samples.  Heparin plasma: Swedish samples |  | Time-resolved fluoroimmunoassay |  | Institute for Preventive Medicine, Nutrition and Cancer, Folkhalsan Research Center, Helsinki, Finland |  | The average of the intra-assay CV was 10.3% |  | The interassay CVs were 11.1% (21 nmol/l), 11.0% (47 nmol/l) and 9.8% (101 nmol/l) |
| MDCS (24) |  | Plasma |  | Time-resolved fluoroimmunoassay |  | Folkhälsan Research Center, Helsinki, Finland |  | The intraassay CV varied from 3.3 to 6.0% in the concentrations from 13.2 to 79.5 nmol/L |  | The interassay CVs varied from 6.9 to 9.9% in concentrations from 16.3 to 96.6 nmol/L |
| NSHDC (25) |  | Serum |  | Time-resolved fluoroimmunoassay |  | Institute for Preventive Medicine, Nutrition and Cancer, Folkhalsan Research Center, Helsinki, Finland |  | - |  | The mean inter-assay CV calculated from these samples was 11.8% |
| Abbreviations: Coefficient of variation (CV), European Prospective Investigation into Cancer and Nutrition (EPIC), Janus Nordic Biological Specimen Biobank Working Group (NBSBWG), Japan Collaborative Cohort Study (JACC), Japan Public Health Center-based prospective Study (JPHC), Malmö Diet and Cancer Study (MDCS), Northern Sweden Health and Disease Cohort (NSHDC). | | | | | | | | | | |

**
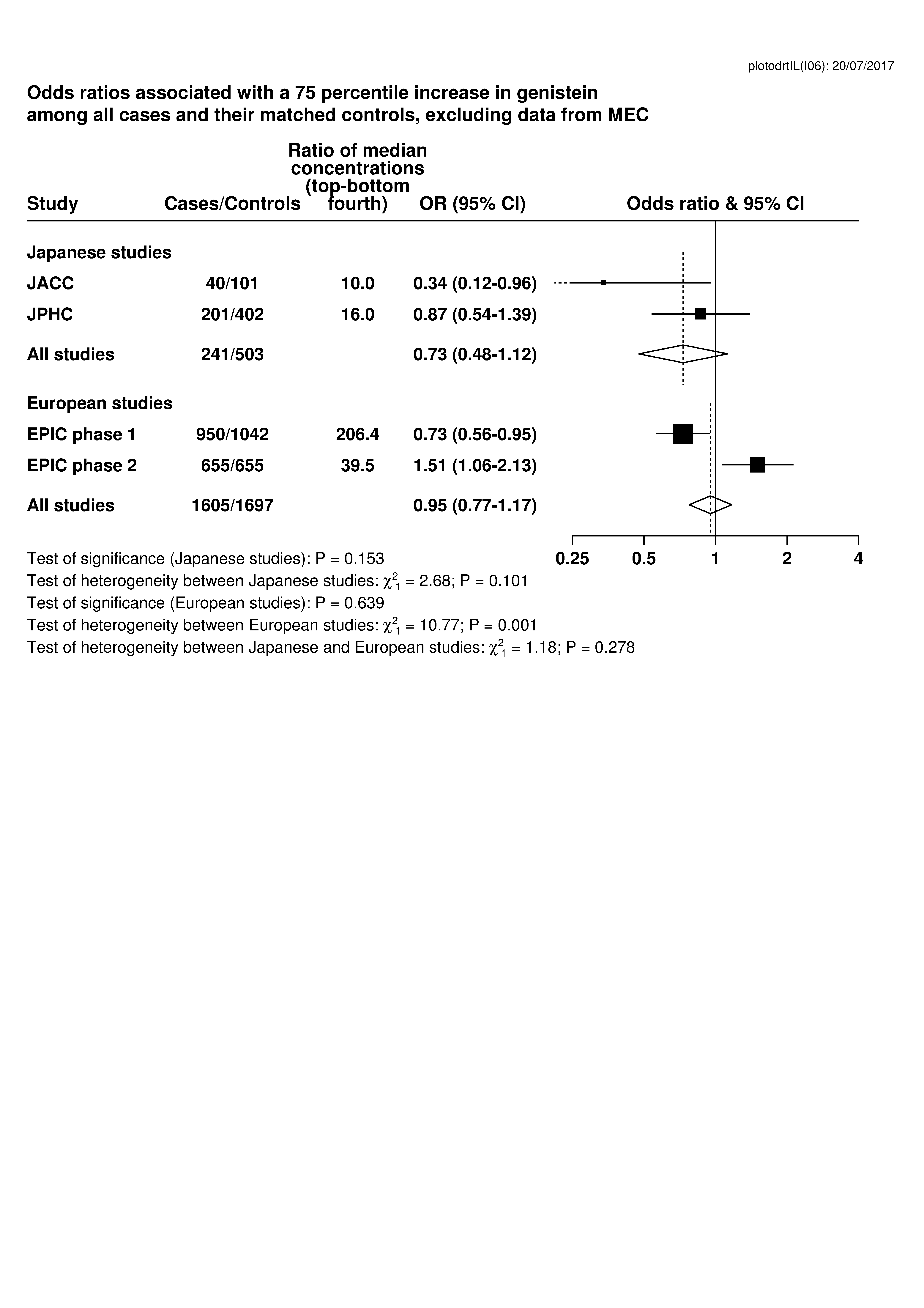
**

**Supplementary Figure 1**. Study-specific ORs (95% CIs) for prostate cancer associated with a 75 percentile increase in genistein concentrations. Estimates are from logistic regression conditioned on the matching variables within each study, but not further adjusted. Heterogeneity in linear trends between studies and between Japanese and European studies was tested by comparing the χ^2^ values for models with and without a (studies) x (linear trend) interaction term. Abbreviations: European Prospective Investigation into Cancer and Nutrition (EPIC), Japan Collaborative Cohort Study (JACC), Japan Public Health Center-based prospective Study (JPHC).

**
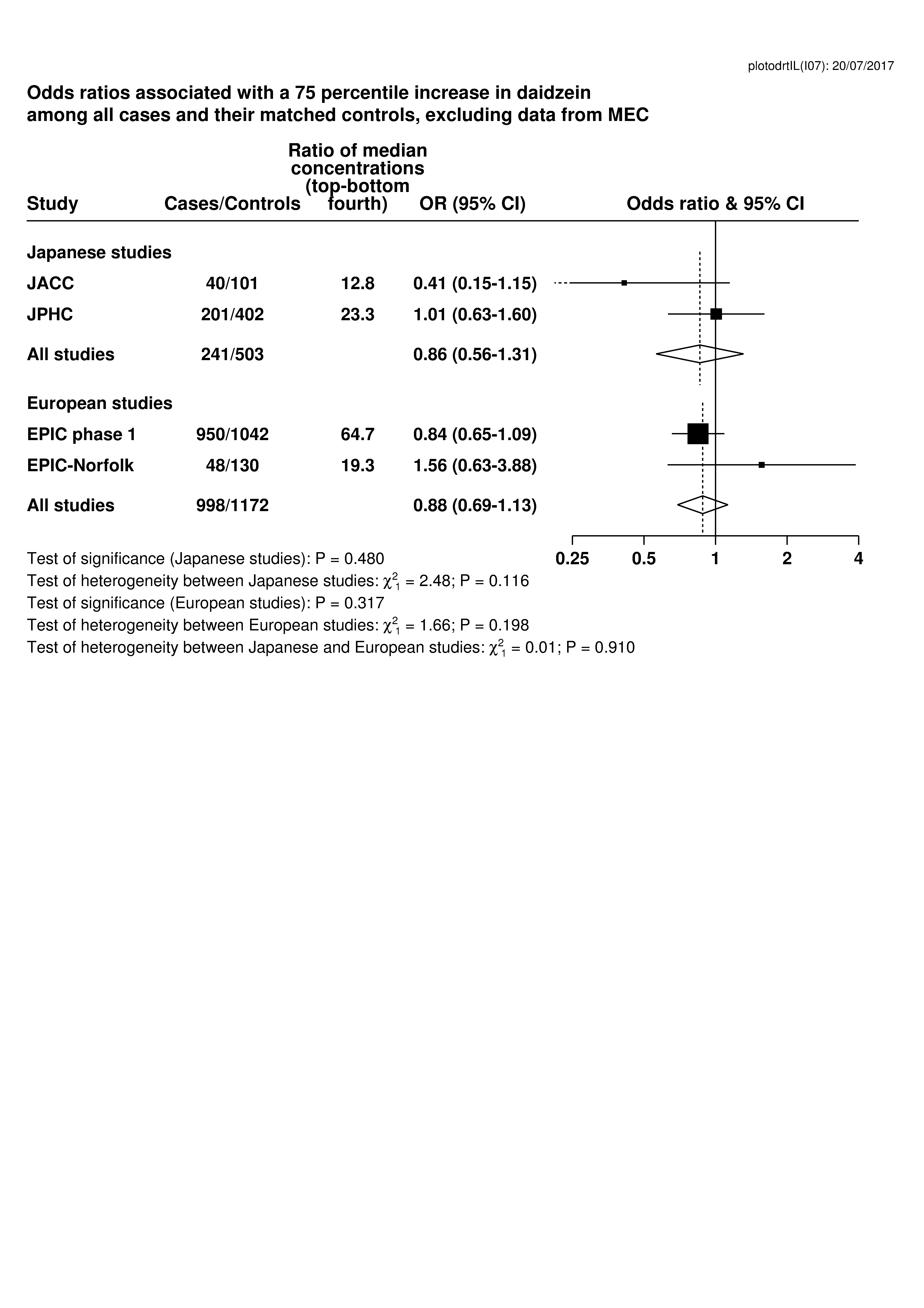
**

**Supplementary Figure 2**. Study-specific ORs (95% CIs) for prostate cancer associated with a 75 percentile increase in daidzein concentrations. Estimates are from logistic regression conditioned on the matching variables within each study, but not further adjusted. Heterogeneity in linear trends between studies and between Japanese and European studies was tested by comparing the χ^2^ values for models with and without a (studies) x (linear trend) interaction term. Abbreviations: European Prospective Investigation into Cancer and Nutrition (EPIC), Japan Collaborative Cohort Study (JACC), Japan Public Health Center-based prospective Study (JPHC).

**
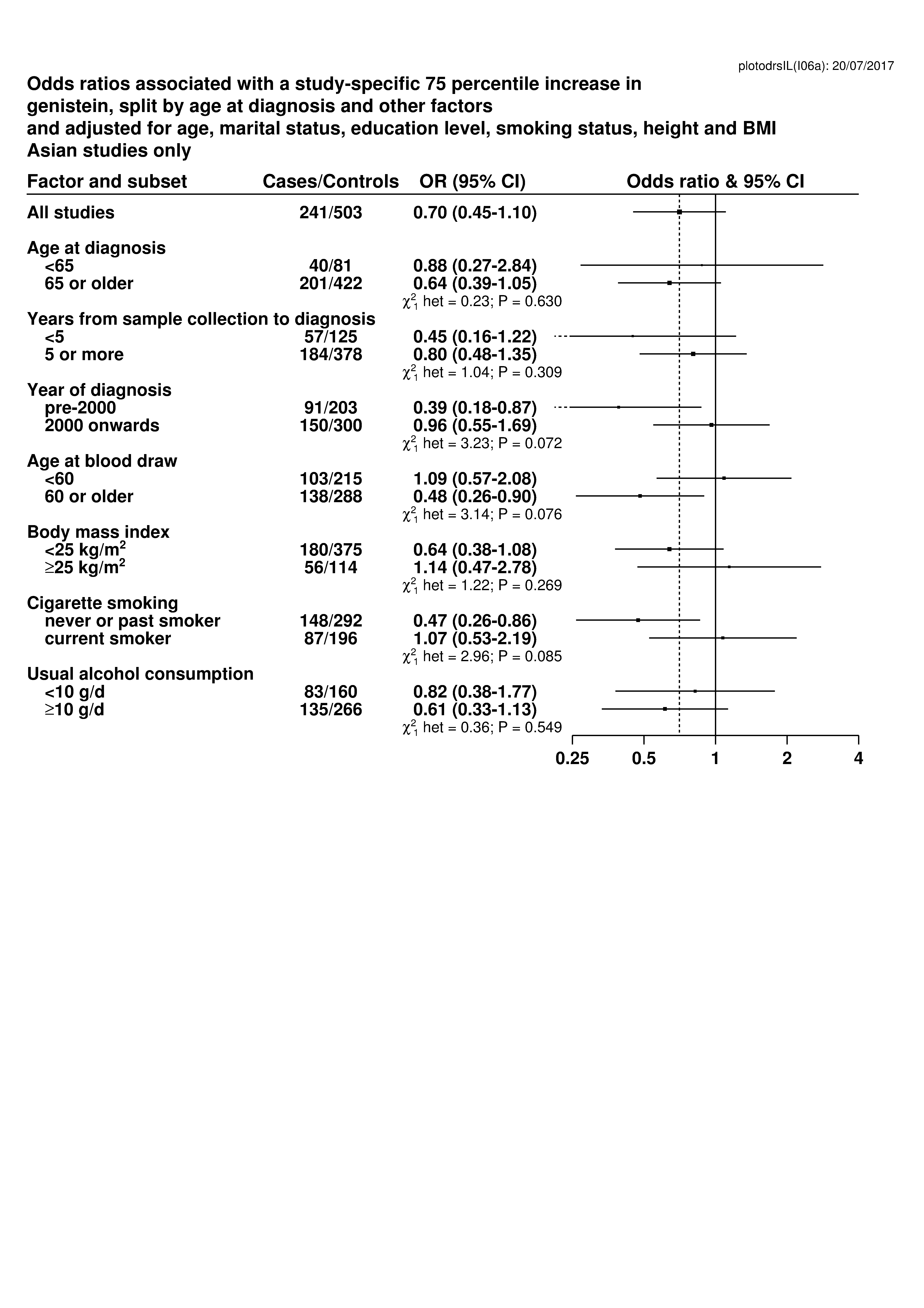
**

**Supplementary Figure 3**. ORs for prostate cancer associated with genistein concentration, according to characteristics of cases and controls in Japanese studies. Each OR is the estimate of the linear trend obtained by replacing the categorical variables representing the fourths of genistein concentration by a continuous variable scored as 0, 0.33, 0.67, and 1. Black squares indicate the OR, and the horizontal lines show the 95% CIs. The area of each square is proportional to the amount of statistical information (inverse of the variance of the logarithm of the OR). The vertical dotted line indicates the OR for all studies. Tests for heterogeneity are for the difference in the association of genistein with prostate cancer risk between subgroups. Estimates are from conditional logistic regression on case-control sets matched within each study and adjusted for age at blood collection (exact), body mass index (BMI; <25, 25–27.4, 27.5–29.9, ≥30 kg/m^2^, unknown), height (≤170, 171–175, 176–180, >180 cm, unknown), marital status (married/cohabiting, not married/cohabiting, unknown), and cigarette smoking (never, past, current, unknown).


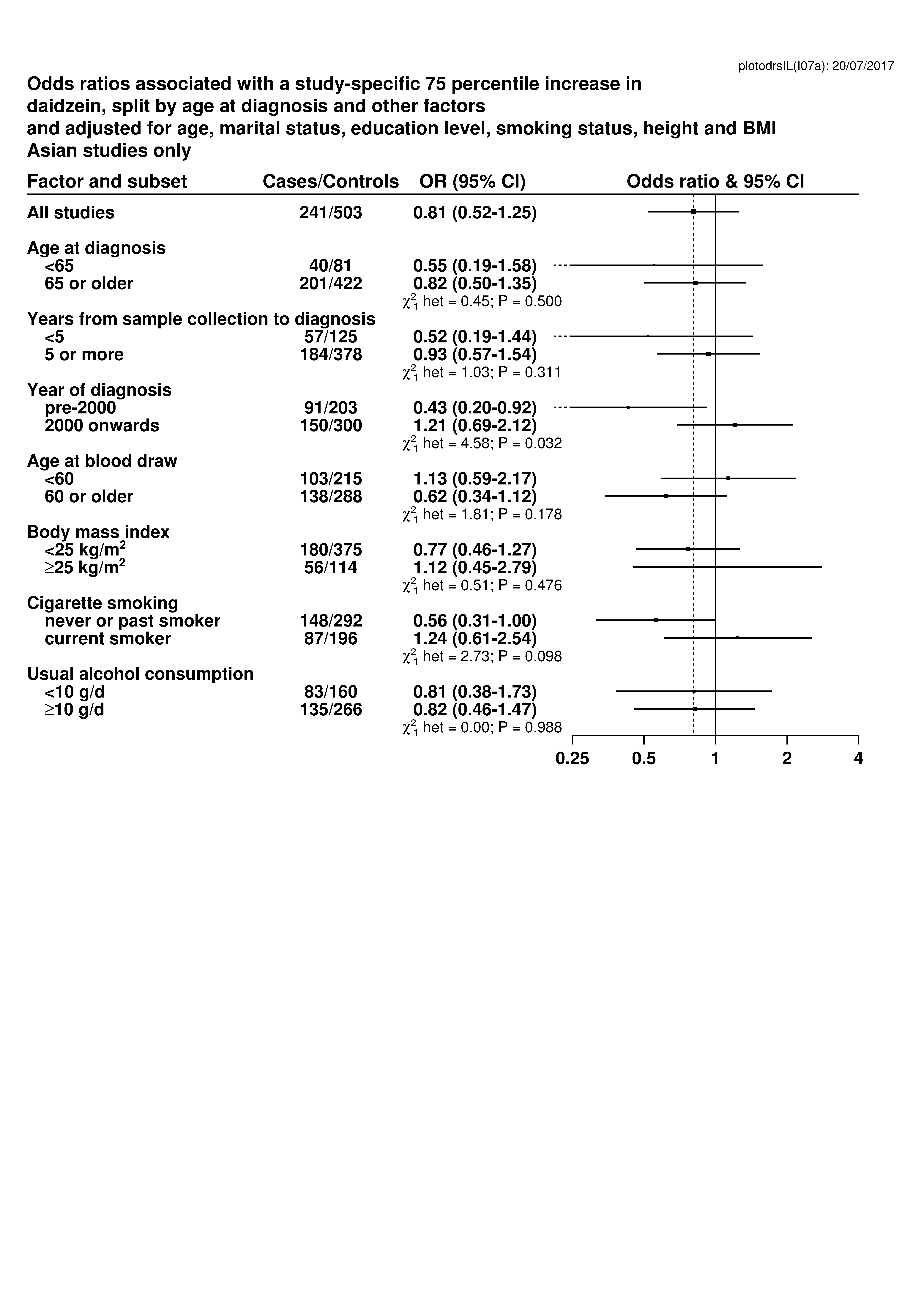


**Supplementary Figure 4**. ORs for prostate cancer associated with daidzein concentration, according to characteristics of cases and controls in Japanese studies. Each OR is the estimate of the linear trend obtained by replacing the categorical variables representing the fourths of daidzein concentration by a continuous variable scored as 0, 0.33, 0.67, and 1. Black squares indicate the OR, and the horizontal lines show the 95% CIs. The area of each square is proportional to the amount of statistical information (inverse of the variance of the logarithm of the OR). The vertical dotted line indicates the OR for all studies. Tests for heterogeneity are for the difference in the association of daidzein with prostate cancer risk between subgroups. Estimates are from conditional logistic regression on case-control sets matched within each study and adjusted for age at blood collection (exact), body mass index (BMI; <25, 25–27.4, 27.5–29.9, ≥30 kg/m^2^, unknown), height (≤170, 171–175, 176–180, >180 cm, unknown), marital status (married/cohabiting, not married/cohabiting, unknown), and cigarette smoking (never, past, current, unknown).

**
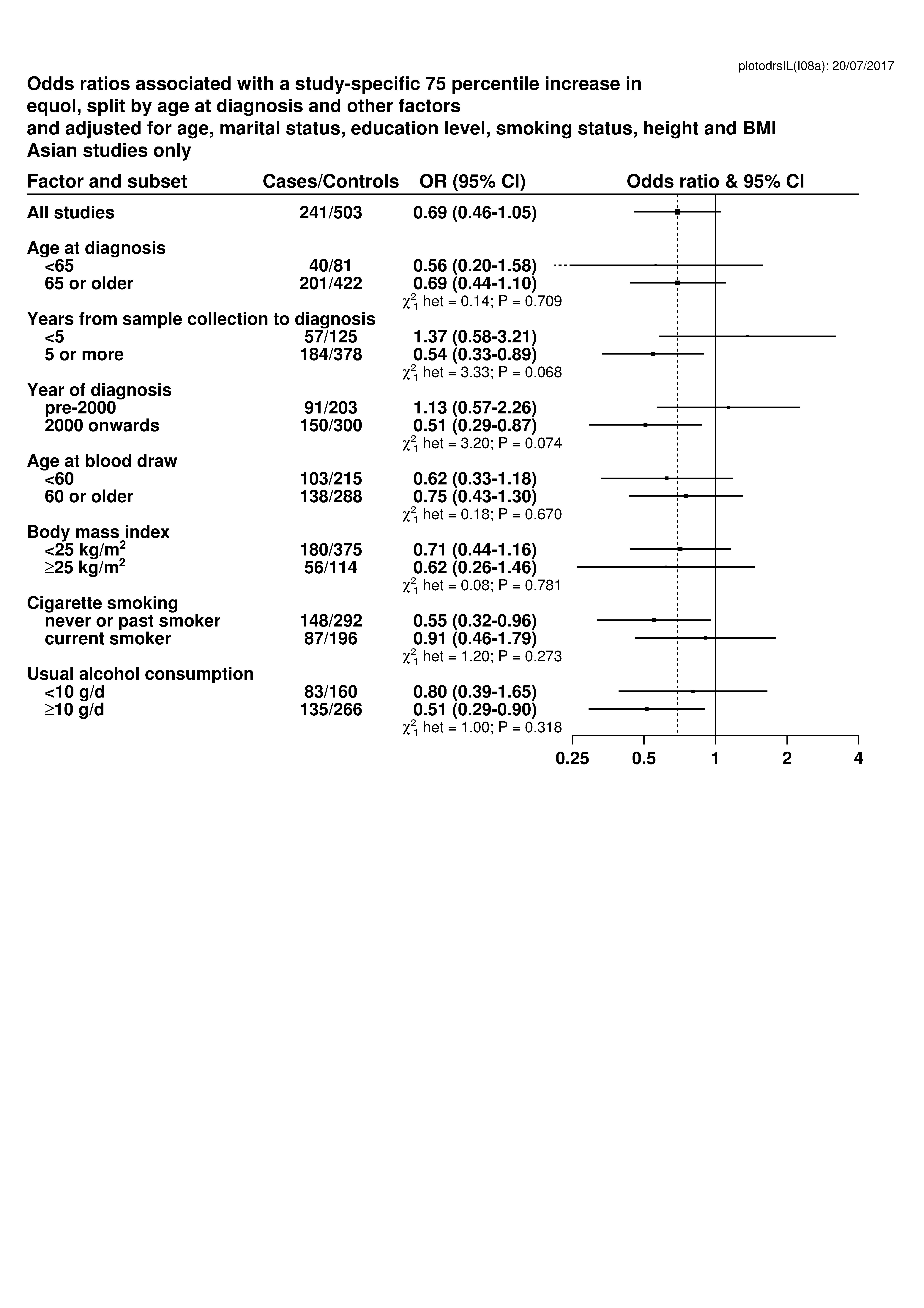
**

**Supplementary Figure 5**. ORs for prostate cancer associated with equol concentration, according to characteristics of cases and controls in Japanese studies. Each OR is the estimate of the linear trend obtained by replacing the categorical variables representing the fourths of equol concentration by a continuous variable scored as 0, 0.33, 0.67, and 1. Black squares indicate the OR, and the horizontal lines show the 95% CIs. The area of each square is proportional to the amount of statistical information (inverse of the variance of the logarithm of the OR). The vertical dotted line indicates the OR for all studies. Tests for heterogeneity are for the difference in the association of equol with prostate cancer risk between subgroups. Estimates are from conditional logistic regression on case-control sets matched within each study and adjusted for age at blood collection (exact), body mass index (BMI; <25, 25–27.4, 27.5–29.9, ≥30 kg/m^2^, unknown), height (≤170, 171–175, 176–180, >180 cm, unknown), marital status (married/cohabiting, not married/cohabiting, unknown), and cigarette smoking (never, past, current, unknown).

**
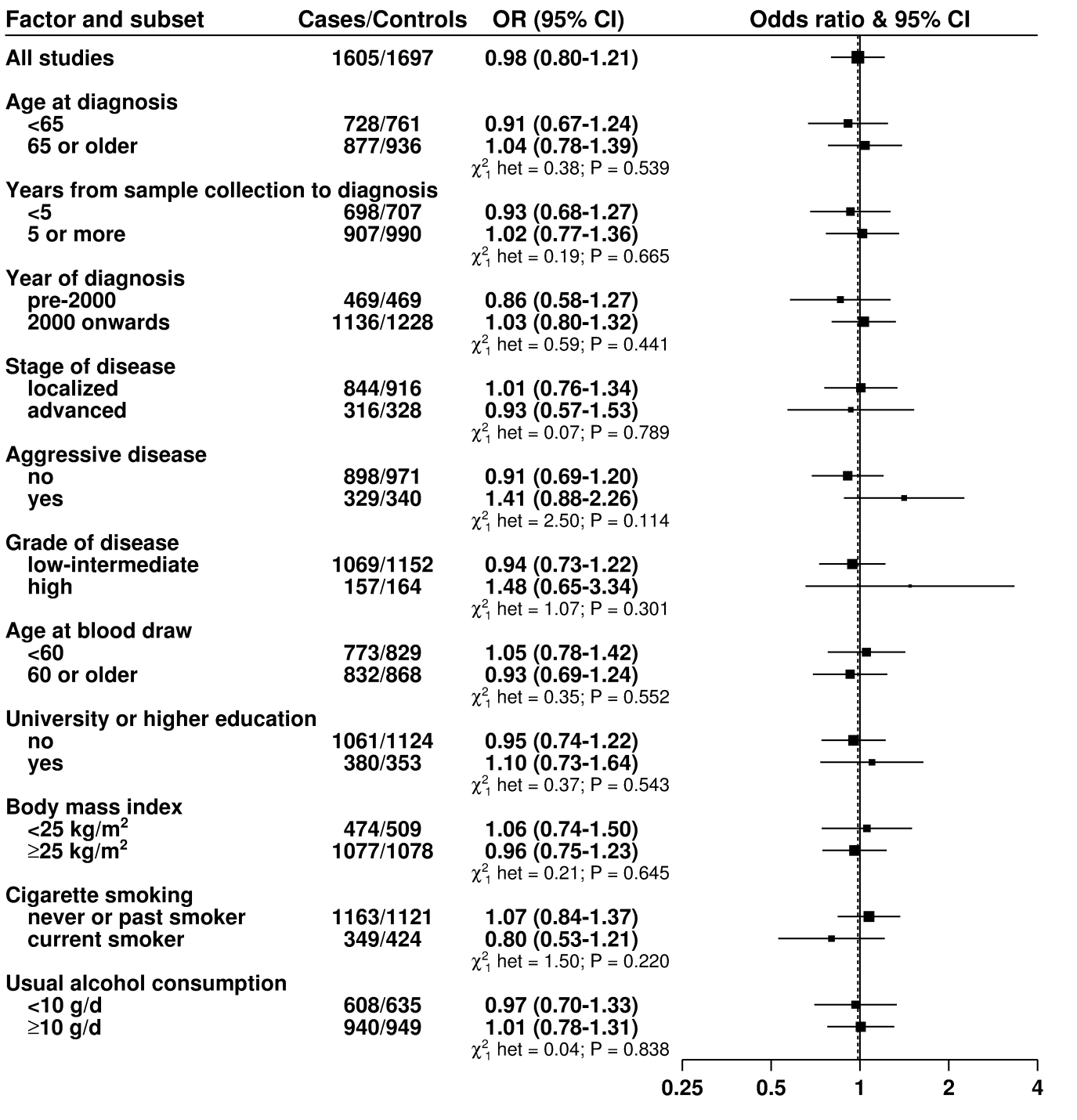
Supplementary Figure 6**. ORs for prostate cancer associated with genistein concentration, according to characteristics of cases and controls in European studies. Each OR is the estimate of the linear trend obtained by replacing the categorical variables representing the fourths of genistein concentration by a continuous variable scored as 0, 0.33, 0.67, and 1. Black squares indicate the OR, and the horizontal lines show the 95% CIs. The area of each square is proportional to the amount of statistical information (inverse of the variance of the logarithm of the OR). The vertical dotted line indicates the OR for all studies. Tests for heterogeneity are for the difference in the association of genistein with prostate cancer risk between subgroups. Estimates are from conditional logistic regression on case-control sets matched within each study and adjusted for age at blood collection (exact), body mass index (BMI; <25, 25–27.4, 27.5–29.9, ≥30 kg/m^2^, unknown), height (≤170, 171–175, 176–180, >180 cm, unknown), marital status (married/cohabiting, not married/cohabiting, unknown), educational status (did not graduate from high school/secondary school/college, high school/secondary school/college graduates, university graduates, unknown), and cigarette smoking (never, past, current, unknown).

**
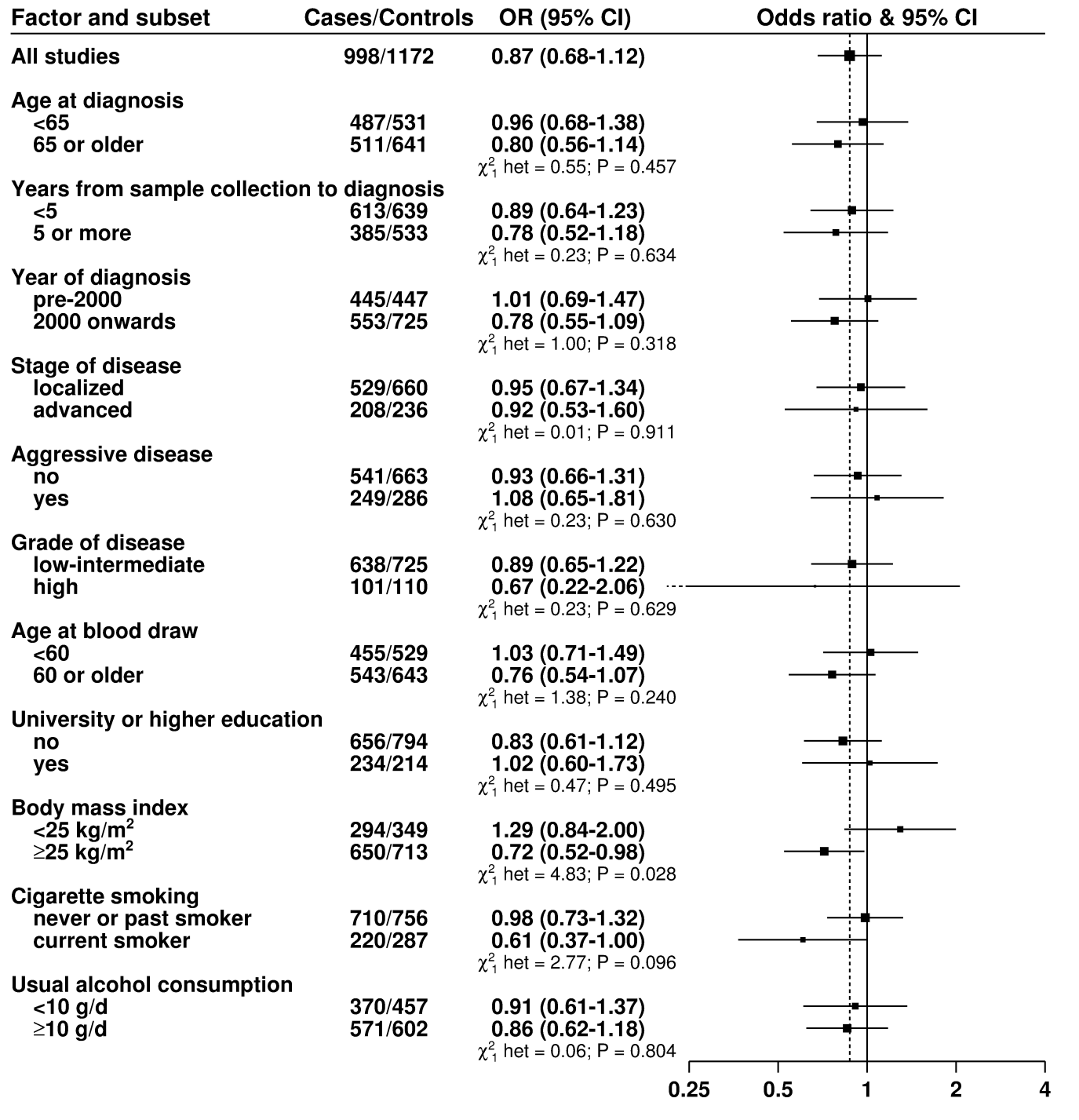
Supplementary Figure 7**. ORs for prostate cancer associated with daidzein concentration, according to characteristics of cases and controls in European studies. Each OR is the estimate of the linear trend obtained by replacing the categorical variables representing the fourths of daidzein concentration by a continuous variable scored as 0, 0.33, 0.67, and 1. Black squares indicate the OR, and the horizontal lines show the 95% CIs. The area of each square is proportional to the amount of statistical information (inverse of the variance of the logarithm of the OR). The vertical dotted line indicates the OR for all studies. Tests for heterogeneity are for the difference in the association of daidzein with prostate cancer risk between subgroups. Estimates are from conditional logistic regression on case-control sets matched within each study and adjusted for age at blood collection (exact), body mass index (BMI = <25, 25–27.4, 27.5–29.9, ≥30 kg/m^2^, unknown), height (≤170, 171–175, 176–180, >180 cm, unknown), marital status (married/cohabiting, not married/cohabiting, unknown), educational status (did not graduate from high school/secondary school/college, high school/secondary school/college graduates, university graduates, unknown), and cigarette smoking (never, past, current, unknown).


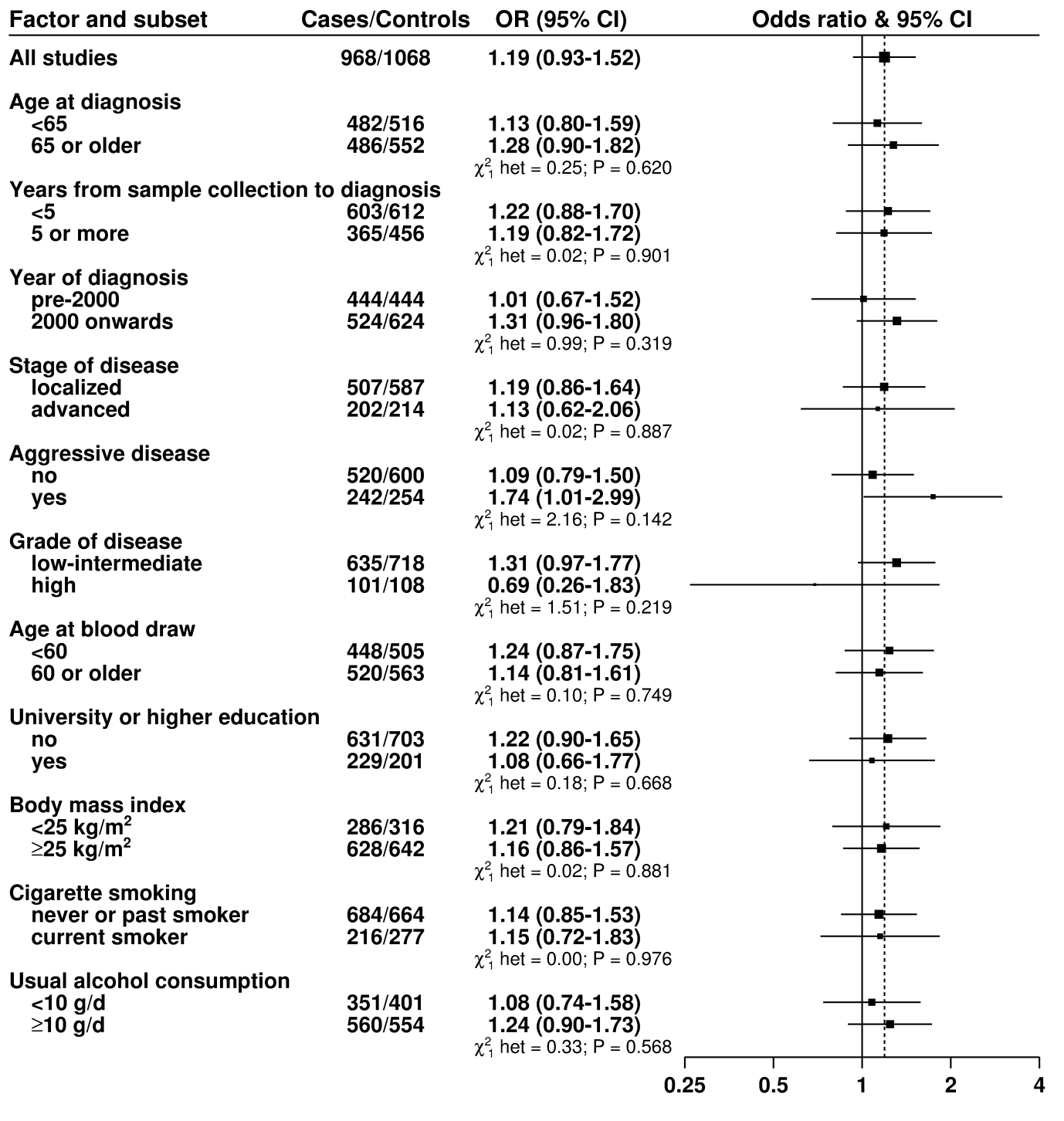
**Supplementary Figure 8**. ORs for prostate cancer associated with equol concentration, according to characteristics of cases and controls in European studies. Each OR is the estimate of the linear trend obtained by replacing the categorical variables representing the fourths of equol concentration by a continuous variable scored as 0, 0.33, 0.67, and 1. Black squares indicate the OR, and the horizontal lines show the 95% CIs. The area of each square is proportional to the amount of statistical information (inverse of the variance of the logarithm of the OR). The vertical dotted line indicates the OR for all studies. Tests for heterogeneity are for the difference in the association of equol with prostate cancer risk between subgroups. Estimates are from conditional logistic regression on case-control sets matched within each study and adjusted for age at blood collection (exact), body mass index (BMI = <25, 25–27.4, 27.5–29.9, ≥30 kg/m^2^, unknown), height (≤170, 171–175, 176–180, >180 cm, unknown), marital status (married/cohabiting, not married/cohabiting, unknown), educational status (did not graduate from high school/secondary school/college, high school/secondary school/college graduates, university graduates, unknown), and cigarette smoking (never, past, current, unknown).

**
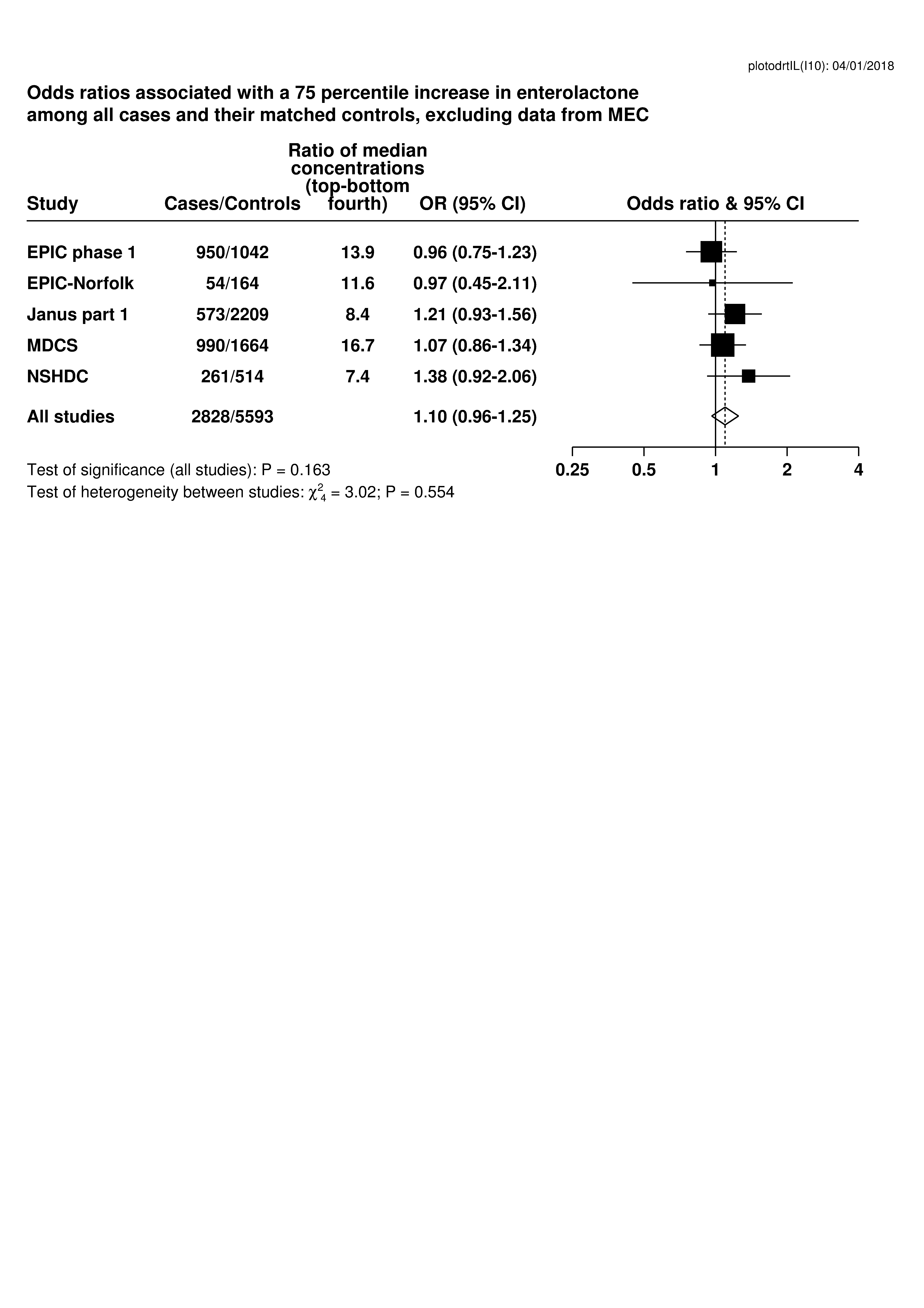
**

**Supplementary Figure 9**. Study-specific ORs (95% CIs) for prostate cancer associated with a 75 percentile increase in enterolactone concentrations. Estimates are from logistic regression conditioned on the matching variables within each study, but not further adjusted. Heterogeneity in linear trends between studies and between Japanese and European studies was tested by comparing the χ^2^ values for models with and without a (studies) x (linear trend) interaction term. Abbreviations: European Prospective Investigation into Cancer and Nutrition (EPIC), Janus Nordic Biological Specimen Biobank Working Group (NBSBWG), the Malmö Diet and Cancer Study (MDCS), Northern Sweden Health and Disease Cohort (NSHDC)

**
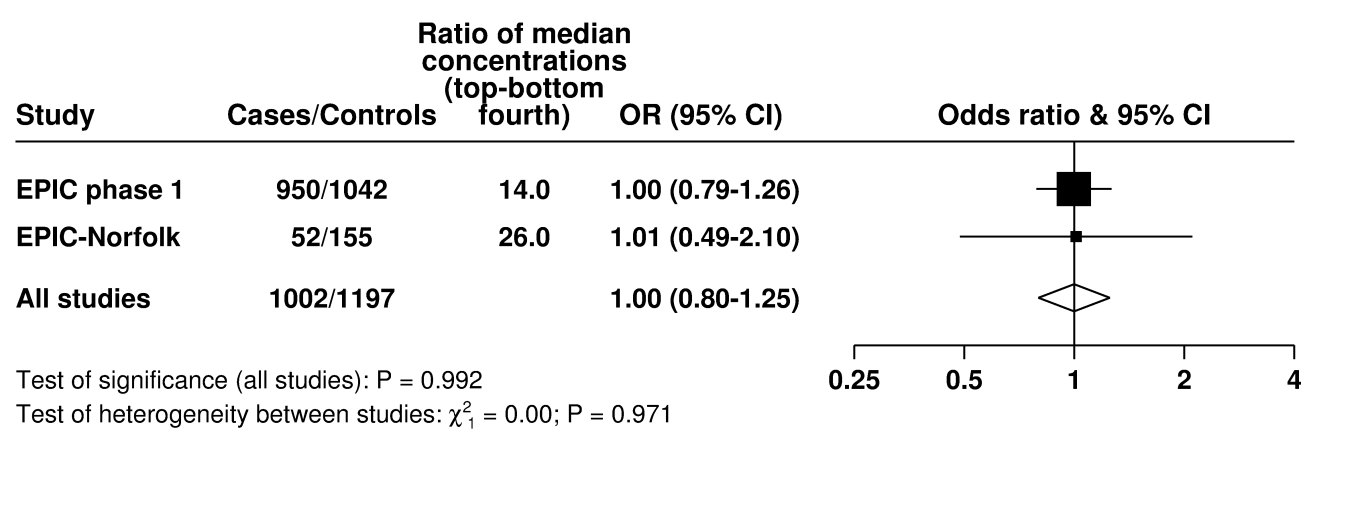
Supplementary Figure 10**. Study-specific ORs (95% CIs) for prostate cancer associated with a 75 percentile increase in enterodiol concentrations. Estimates are from logistic regression conditioned on the matching variables within each study, but not further adjusted. Heterogeneity in linear trends between studies and between Japanese and European studies was tested by comparing the χ^2^ values for models with and without a (studies) x (linear trend) interaction term. Abbreviation: European Prospective Investigation into Cancer and Nutrition (EPIC).

**
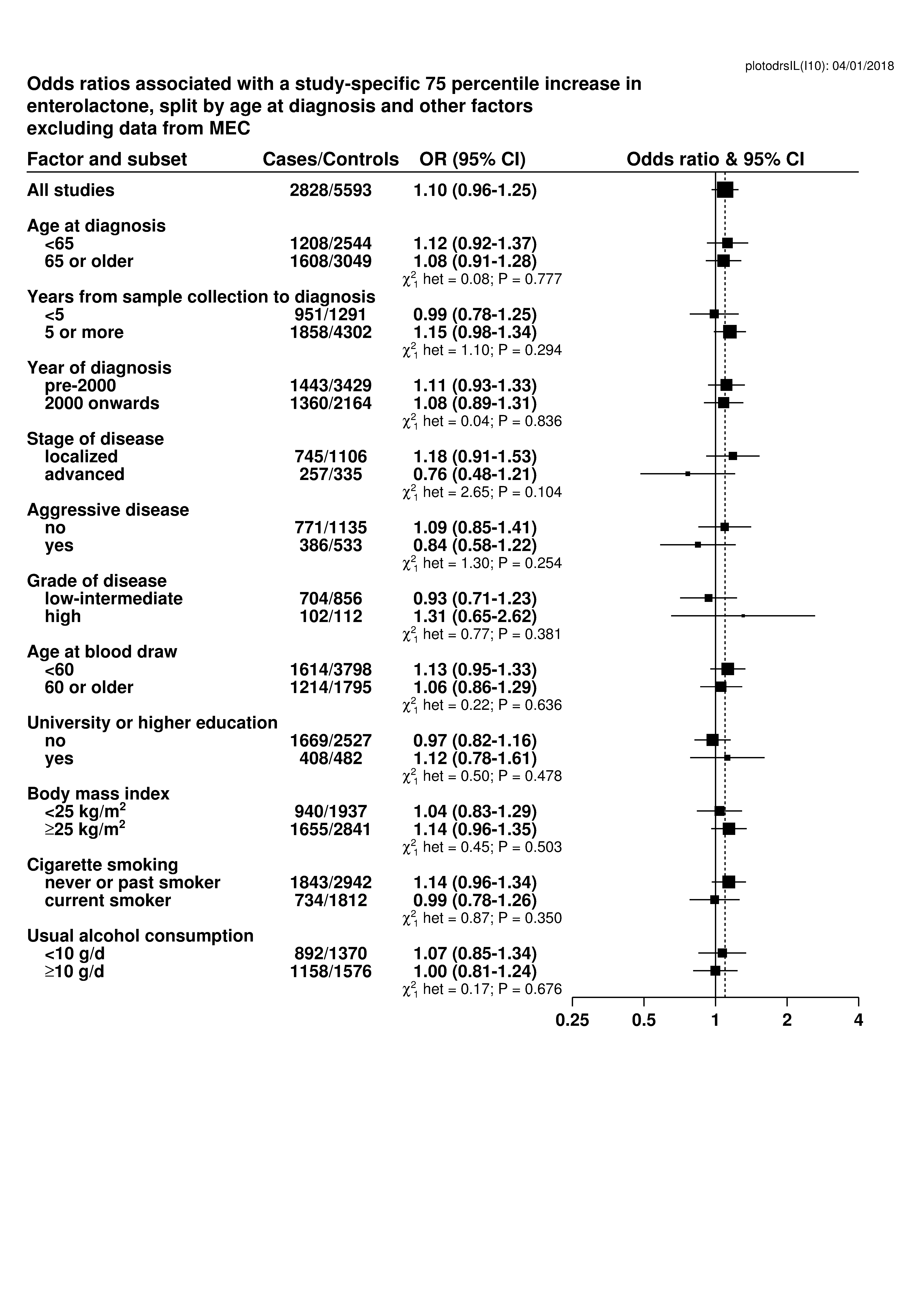
**

**Supplementary Figure 11**. ORs for prostate cancer associated with enterolactone concentration, according to characteristics of cases and controls. Each OR is the estimate of the linear trend obtained by replacing the categorical variables representing the fourths of enterolactone concentration by a continuous variable scored as 0, 0.33, 0.67, and 1. Black squares indicate the OR, and the horizontal lines show the 95% CIs. The area of each square is proportional to the amount of statistical information (inverse of the variance of the logarithm of the OR). The vertical dotted line indicates the OR for all studies. Tests for heterogeneity are for the difference in the association of enterolactone with prostate cancer risk between subgroups. Estimates are from conditional logistic regression on case-control sets matched within each study, but not further adjusted.

**
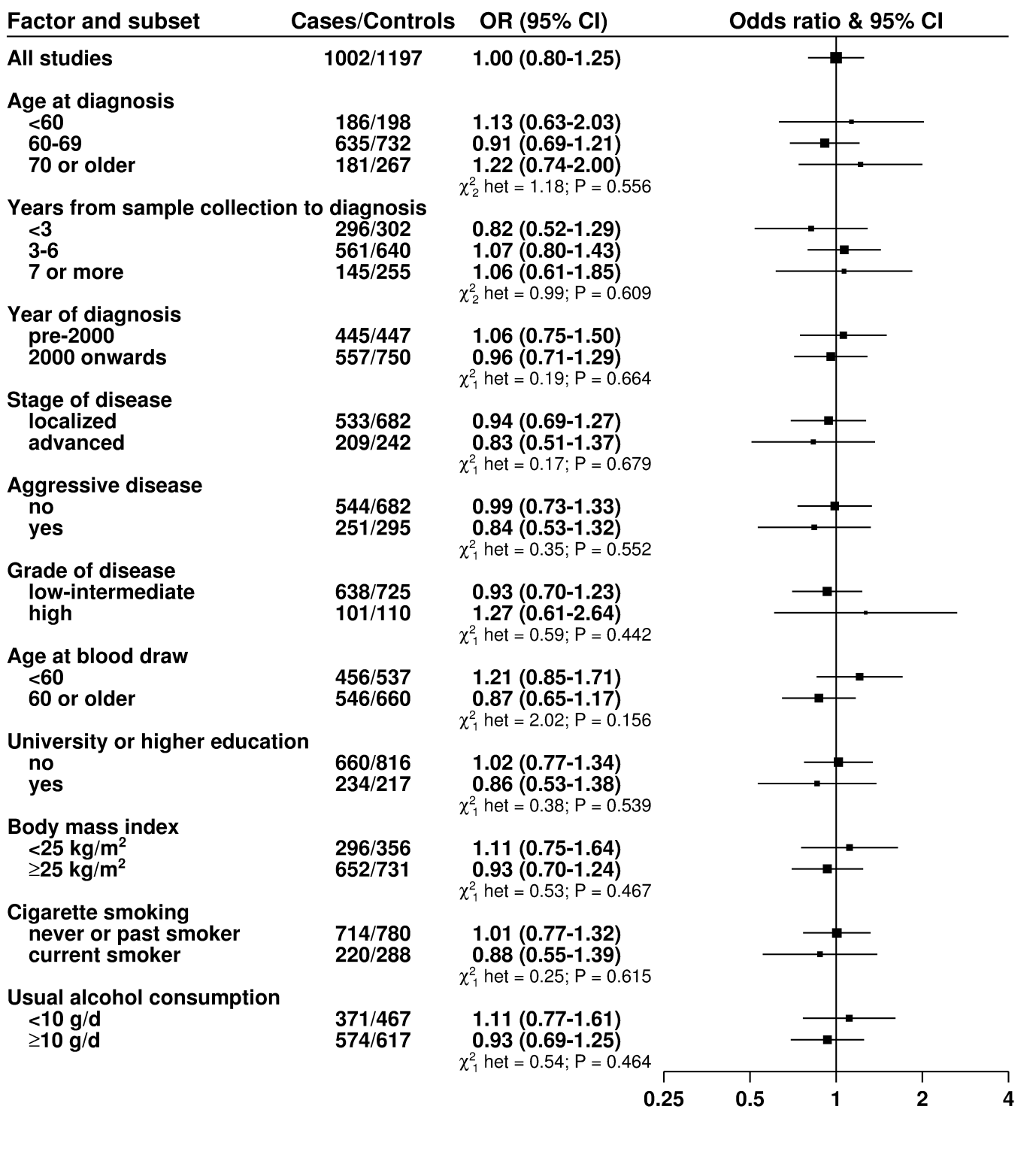
**

**Supplementary Figure 12**. ORs for prostate cancer associated with enterodiol concentration, according to characteristics of cases and controls. Each OR is the estimate of the linear trend obtained by replacing the categorical variables representing the fourths of enterodiol concentration by a continuous variable scored as 0, 0.33, 0.67, and 1. Black squares indicate the OR, and the horizontal lines show the 95% CIs. The area of each square is proportional to the amount of statistical information (inverse of the variance of the logarithm of the OR). The vertical dotted line indicates the OR for all studies. Tests for heterogeneity are for the difference in the association of enterodiol with prostate cancer risk between subgroups. Estimates are from conditional logistic regression on case-control sets matched within each study, but not further adjusted.
